# Dietary cholesterol directly induces acute inflammasome-dependent intestinal inflammation

**DOI:** 10.1038/ncomms6864

**Published:** 2014-12-23

**Authors:** Fränze Progatzky, Navjyot J. Sangha, Nagisa Yoshida, Marie McBrien, Jackie Cheung, Alice Shia, James Scott, Julian R. Marchesi, Jonathan R. Lamb, Laurence Bugeon, Margaret J. Dallman

**Affiliations:** 1 Department of Life Sciences, Faculty of Natural Sciences, Imperial College London, Sir Alexander Fleming Building, London SW7 2AZ, UK; 2 National Heart and Lung Institute, Imperial College London, London W12 0NN, UK; 3 Computational and Systems Medicine, Faculty of Medicine, Department of Surgery and Cancer, Imperial College London, London SW7 2AZ, UK; 4 Section of Hepatology, Imperial College London, Norfolk Place, London W2 1NY, UK; 5 Centre for Digestive and Gut Health, Imperial College London, London W2 1NY, UK; 6 School of Biosciences, Cardiff University, Museum Avenue, Cardiff CF10 3AX, UK

## Abstract

Prolonged ingestion of a cholesterol- or saturated fatty acid-enriched diet induces chronic, often systemic, auto-inflammatory responses resulting in significant health problems worldwide. *In vivo* information regarding the local and direct inflammatory effect of these dietary components in the intestine and, in particular, on the intestinal epithelium is lacking. Here we report that both mice and zebrafish exposed to high-fat (HFDs) or high-cholesterol (HCDs) diets develop acute innate inflammatory responses within hours, reflected in the localized interleukin-1β-dependent accumulation of myeloid cells in the intestine. Acute HCD-induced intestinal inflammation is dependent on cholesterol uptake via Niemann-Pick C1-like 1 and inflammasome activation involving apoptosis-associated Speck-like protein containing a caspase recruitment domain, which leads to Caspase-1 activity in intestinal epithelial cells. Extended exposure to HCD results in localized, inflammation-dependent, functional dysregulation as well as systemic pathologies. Our model suggests that dietary cholesterol initiates intestinal inflammation in epithelial cells.

## Abstract (web summary)

Chronic consumption of a Western-type diet leads to systemic inflammation of undefined origin, which contributes to metabolic disease. Here Progatzky *et al*. identify an immediate early step in the process by showing that dietary cholesterol rapidly activates inflammasomes in the gut epithelium.

## Graphical abstract

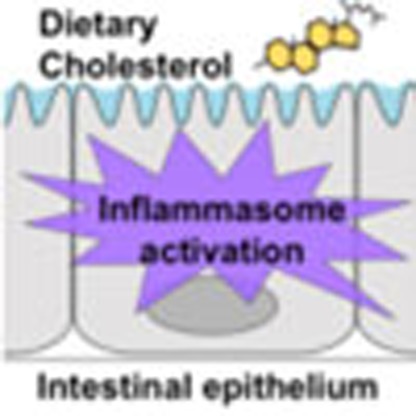

Over the past decade, an increase in the consumption of Western-type diets rich in high-fat/cholesterol, high-protein and high-sugar has been observed in the Western world coinciding with the occurrence in inflammatory bowel disease (IBD) and other systemic immune-related human disorders^1^. A systematic review by Hou *et al*.^2^ of 19 studies has found an association between Western-type diets and the increased risk of ulcerative colitis and Crohn’s disease. In mice, recent evidence has shown that prolonged exposure to high-fat diet (HFD) can indirectly induce intestinal inflammation by altering the microbiota and perturbing immune homeostasis^3^,^4^. However, mechanistic evidence regarding any direct effect of acute exposure to dietary fats on intestinal responses *in vivo* is lacking, despite the theoretical concept of fatty-acid-induced inflammation suggested by *in vitro* studies using intraepithelial lymphocytes^5^ and intestinal epithelial cells^6^. The intestinal mucosa is the first barrier where fat is encountered, absorbed and metabolized, and might therefore be involved in responses triggered by dietary lipids. Both resident antigen-presenting cells and intestinal mucosal epithelial cells are equipped with innate immune sensors, the pattern recognition receptors (PRR), which can detect conserved molecular features specific to microbes, to defend the organism from harmful pathogens and promote repair, regeneration and intestinal immune homeostasis^7^,^8^,^9^,^10^. These receptors are now known to bind to damage associated molecular patterns as well. Recent findings have demonstrated that fatty acids and cholesterol are potent ligands for these receptors and lead to inflammasome activation in haematopoietic cells *in vitro*^11^,^12^. Whether these dietary components or food antigens can directly activate inflammasomes in the intestine and induce inflammatory processes *in vivo* is an important but unanswered question.

In this study we have chosen zebrafish as our primary experimental organism, because in addition to genetic tractability and conservation of immune and inflammatory pathways^13^,^14^, their optical translucency allows an integrative multi-organ analysis of the pathophysiological outcomes of ingestion of HFDs or high-cholesterol diets (HCDs). As proteins involved in the transport of dietary fat and lipids in zebrafish, including the ezetimibe-sensitive cholesterol-binding protein, Niemann-Pick C1-like 1 (NPC1L1), are conserved with those in mammals, zebrafish is a relevant model for the study of dietary fat and cholesterol uptake and processing^15^,^16^,^17^.

Here we report that both mice and zebrafish exposed to HFDs or HCDs respond within hours with a localized accumulation of myeloid cells in the intestine. Through treatment of zebrafish with selective pharmacological inhibitors and by using germ-free (GF) larvae, we demonstrate that acute HCD-induced myeloid cell accumulation is primarily and directly dependent on cholesterol uptake by NPC1L1 and secondarily dependent on constitutive PRR and nuclear factor-κB (NF-κB) activation by the commensal microbiota. These combined signals lead to Caspase-1 activation in intestinal epithelial cells. Inflammasome activation following HCD was further confirmed using a novel approach to deliver morpholino (MO) oligonucleotides through which knockdown of apoptosis-associated Speck-like protein containing a caspase recruitment domain (ASC) and interleukin 1 β (IL-1β) abrogates HCD-induced intestinal myeloid cell accumulation. Extending the HCD exposure to 10 days results in local pathologies also directly dependent on cholesterol binding/uptake and inflammasome activation.

## Results

### HFD induces myeloid cell accumulation in the intestine

**Figure 1:**
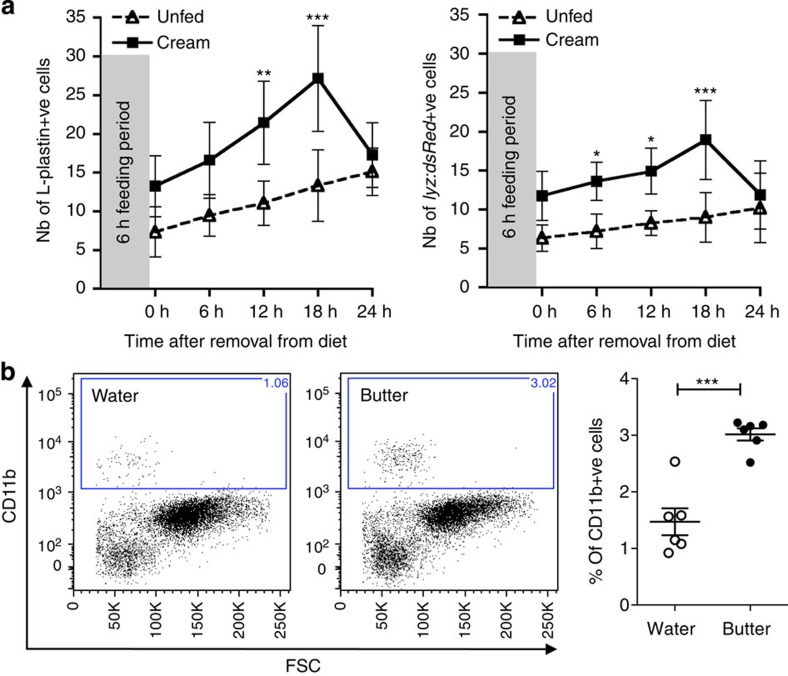
HFD induces myeloid cell accumulation in the intestine. (**a**) Total number of L-plastin+ cells in WT and dsRed+ cells in *Tg(lyz:dsRed)* larval intestines at 6 dpf left unfed (triangles) or fed cream (squares) for 6 h. One representative experiment of at least two with *n*≥15 is shown. Error bars represent 95% confidence intervals. Two-way analysis of variance. (**b**) Representative flow cytometry plots and quantification of CD11b labelling of cells in the small intestine (distal jejunum and ileum) of Balb/C mice 12 h after water or butter gavage. Each dot represents one individual mouse pooled from two experiments (*n*=6). Mann–Whitney test. Error bars: s.e.m. ***P<0.001, **P<0.01 and *P<0.05.

Schlegel *et al*.^18^ have shown that dietary lipids, in the form of cream high in fat and cholesterol, were efficiently absorbed after 6 h feeding in larval zebrafish. We therefore first tested the effects of a short exposure to such a diet on intestinal inflammation in zebrafish. Following a single short (6 h) exposure to cream (see Supplementary Fig. 1 for treatment protocols), we observed a significant, transient increase in the number of L-Plastin-labelled myeloid cells^19^ and dsRed+ neutrophils in the intestine of wild type (WT) and *Tg(lyz:dsRed)*^20^ larvae, respectively. Both cell types were already enriched in the intestines of cream fed fish (compared with unfed fish) by the end of the 6-h feeding period, while the peak of myeloid cell accumulation was at 18 h post feeding (Fig. 1a). We validated these initial findings by exposing mice to a similar HFD in the form of melted butter and recorded an increased accumulation of CD11b+ myeloid cells in the small intestine with similar kinetics to that observed in zebrafish (Fig. 1b). These data confirmed that the acute intestinal inflammation occurring in response to HFD in zebrafish is shared with mammals.

### HCD induces myeloid cell accumulation in the intestine

**Figure 2:**
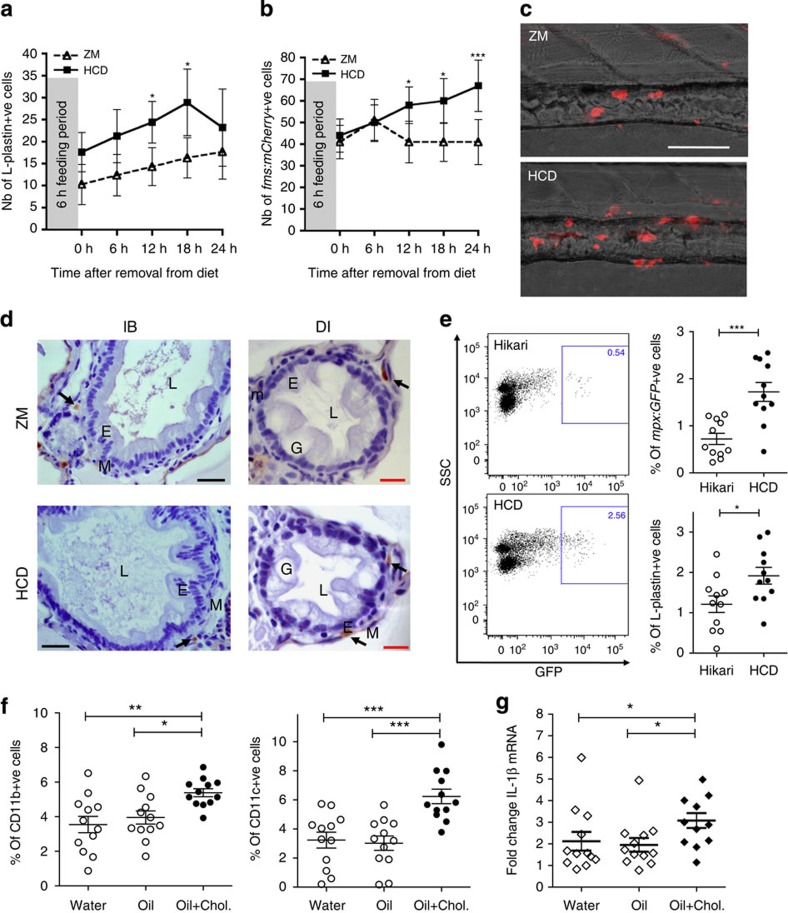
HCD induces myeloid cell accumulation in the intestine. (**a**) Total number of L-plastin+ cells in the intestine of 6 dpf WT larvae following HCD (squares) or ZM (triangles) for 6 h. Two-way analysis of variance (ANOVA). (**b**) Total number of mCherry+ cells in the intestine of *Tg(fms:mCherry)* larvae following HCD or ZM for 6 h. In **a** and **b**, one representative experiment of at least two with *n*≥15 is shown. Error bars represent 95% confidence intervals. Two-way ANOVA. (**c**) Representative images of distal intestine of *Tg(fms:mCherry)* larvae after 18 h following ZM or HCD for 6 h. Scale bar, 100 μm. (**d**) L-plastin+ cells (arrow) localized to the muscularis of intestine layers; intestinal bulb (IB), distal intestine (DI), lumen (L), goblet cells (G), epithelial layer (E), muscularis (M). Scale bars, 20 μm (black); 10 μm (red). (**e**) Representative flow cytometry plots and quantification of GFP+ and L-Plastin+ cells of adult *Tg(mpx:GFP)* intestine tissue after 15 h following HCD or Hikari control for 6 h. Each dot represents one individual fish pooled from three experiments (*n*=11). Two-tailed *t*-test. One-way ANOVA. Error bars are s.e.m. (**f**) CD11b+ and CD11c+ cells in the small intestine (distal jejunum and ileum) of Balb/C mice 12 h after gavage with water, oil or oil+cholesterol. One-way ANOVA. Error bars are s.e.m. (**g**) Fold change of IL-1β mRNA assessed qRT–PCR in the small intestines (ileum) of Balb/C mice. Relative expression values were normalised to 18S and expressed relative to one control sample. Kruskal–Wallis test. Error bars are s.e.m. In **f** and **g**, each dot represents one individual mouse and data are pooled from four experiments (*n*=12). ***P<0.001, **P<0.01 and *P<0.05.

Because of the reported pro-inflammatory effects of cholesterol in a variety of tissue pathologies^12^,^21^,^22^, we refined the inflammatory challenge by introducing cholesterol into the standard zebrafish diet, ZM, and compared the effect of the cholesterol-enriched diet (HCD) with unmodified ZM. The HCD resulted in an accumulation of L-Plastin+ cells in the intestine (Fig. 2a) in a dose-dependent manner (Supplementary Fig. 2a). The kinetics of intestinal inflammatory cell accumulation following the HCD were similar to those induced by cream (comparing Figs 1a and 2a). We observed similar kinetics, albeit with lower levels of accumulation of neutrophils, marked in WT fish by staining for myeloperoxidase (MPO)^23^ or using *Tg(lyz:dsRed)*, or myeloid precursors, fluorescently labelled in *Tg(pu.1:GFP)*^24^ larvae (Supplementary Fig. 2b), suggesting the induction of generalized myeloid cell inflammation. Macrophages, as observed in *Tg(fms:mCherry)* fish^25^, accumulated significantly by 12 h following the 6-h HCD feeding and this accumulation did not resolve by 24 h (Fig. 2b,c). These results show that a single short feed of HCD can induce significant accumulation of myeloid cells, manifest by an increase of neutrophils followed by macrophages, reminiscent of other models of inflammation^25^. No immune cell accumulation was detected when comparing unfed with ZM control diet-fed larvae, corroborating that the cholesterol is the inflammatory component of the diet (Supplementary Fig. 2c). Histological examination revealed that L-Plastin+ myeloid cells in the distal intestine and the intestinal bulb were found in the muscularis layer of the larval gut below the epithelial layer (Fig. 2d) and this localization was not affected by the HCD. Feeding of adult *Tg(mpx:GFP)*^26^ fish with HCD revealed a similar accumulation of both GFP+ neutrophils and L-Plastin+ haematopoietic cells (Fig. 2e), showing that cholesterol-induced inflammation also occurred in fully immunocompetent adult fish. We again validated these findings by exposing mice to HCD. As fatty acids are required for cholesterol uptake^16^, mice gavaged with water were compared with those gavaged with corn oil alone or corn oil supplemented with cholesterol using an amount that corresponds to a day feed of 0.2% cholesterol (the percentage used routinely in atherogenic diets in mice^27^). We found an increased accumulation of CD11b+ and CD11c+ myeloid cells in the small intestine after 12 h following gavage of oil+cholesterol but not oil alone when compared with water (Fig. 2f). This result confirmed that acute intestinal inflammation occurs in response to high cholesterol rather than fat alone, at least under these conditions. Moreover, although no significant changes in expression of NLRP3, NLRP6, NLRC4 or IL-18 messenger RNA were detected (Supplementary Fig. 3), we found a small, but significant increase of IL-1β transcripts after 12 h in isolates of the small intestine in mice gavaged with oil+cholesterol compared with controls (Fig. 2g).

### HCD-induced inflammation is dependent on NPC1L1 and NF-κB

**Figure 3:**
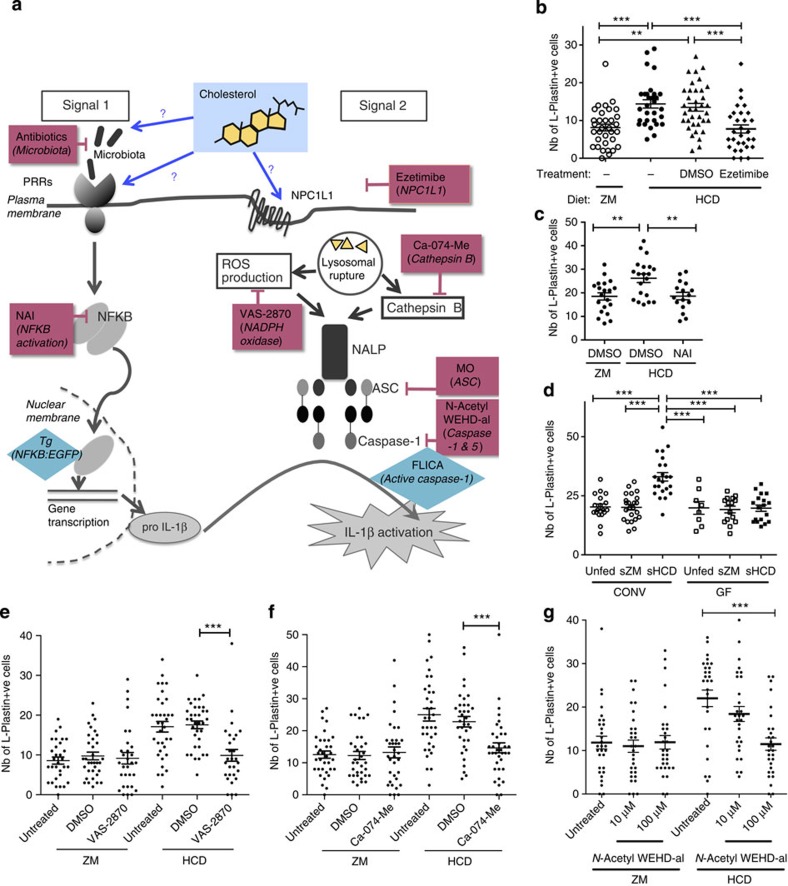
HCD-induced myeloid cell accumulation is dependent on cholesterol binding and inflammasome activation. (**a**) Schematic representation of the two signal model of activation of the inflammasome. Magenta boxes indicate chemical or genetic tools (MO) used and turquoise diamonds indicate reporter activity tools. (**b**) Total number of intestinal L-Plastin+ cells in ezetimibe (25 μM) or DMSO-treated larvae. *n*≥30, one representative experiment of three. One-way analysis of variance (ANOVA). (**c**) Total number of intestinal L-plastin+ cells in NAI inhibitor (150 nM)-treated larvae. *n*≥16, one representative experiments of two. One-way ANOVA. (**d**) Total number of intestinal L-plastin+ cells in larvae reared in conventional (CONV) or GF conditions and fed sterile HCD, ZM control diet or unfed. *n*≥8, pooled from two experiments. One-way ANOVA. (**e**) Total number of intestinal L-plastin+ cells in NADPH oxidase inhibitor VAS-28701 (1 μM)-treated larvae. *n*≥28, pooled from two experiments. Kruskal–Wallis test. (**f**) Total number of intestinal L-plastin+ cells in Cathepsin B inhibitor Ca-074-Me (100 μM)-treated larvae. *n*≥34, pooled from two experiments. Kruskal–Wallis test. (**g**) Total number of intestinal L-Plastin+ cells in Caspase-1 inhibitor *N*-AcetylWEHD-al-treated larvae. *n*≥29, pooled from two experiments. Kruskal–Wallis test. From **b** to **g**, each dot represents one individual larva. Error bars are s.e.m. ***P<0.001 and **P<0.01.

Cholesterol, oxidized low-density lipoprotein and saturated fatty acids activate the NALP3 inflammasome in mammalian macrophages^11^,^12^. Although components of the inflammasome are present in other cells, including the intestinal epithelium^7^,^8^, it is not known whether or not cholesterol can trigger its activation in those cells. Inflammasome activation requires two distinct signals: signal 1 activated by inflammatory stimuli such as tumour necrosis factor or PRR-mediated signalling, resulting in pro-inflammatory cytokine production, and signal 2, which results in Caspase-1 activation and cytokine processing (Fig. 3a). Before we targeted various components of these signalling pathways, we assessed the role of the cholesterol-binding protein NPC1L1 in inflammation. As HCD exposure led to the accumulation of many myeloid lineages around the intestine, we used the pan-myeloid marker, L-Plastin, to assess myeloid cell accumulation following HCD in all further experiments in zebrafish larvae. Treatment with ezetimibe, which inhibits cholesterol binding/uptake via NPC1L1 (ref. 15), abolished the accumulation of myeloid cells induced with HCD (Fig. 3b). Ezetimibe did not affect food intake or transit, as assessed using food supplemented with fluorescently labelled microspheres in control experiments (Supplementary Figs 4b and 5b). These results show that binding/uptake of cholesterol via NPC1L1, rather than its presence in the gut lumen, was essential to mediate its inflammatory effects.

To target NF-κB, the central pathway of signal 1, we used NAI, an inhibitor of NF-κB activation^28^, and found that the accumulation of L-Plastin+ myeloid cells consequent to the HCD was abrogated (Fig. 3c). The effect of NAI on myeloid cell accumulation was not due to a change in food intake or intestinal transit, as NAI treatment up to doses of 150 nM did not affect the amount of food uptake or food transit rates (Supplementary Figs 4c and 5c).

### The microbiota is required for HCD-induced inflammation

It has been shown that the commensal microbiota can activate PRRs and NF-κB in the intestine^29^,^30^, therefore potentially providing signal 1 of inflammasome activation even in the steady state^31^. To assess the role of commensal microbes in provision of signal 1, germ-free fish were raised and fed sterile HCD (sHCD). We found that unlike control fish, conventionally raised and fed sterile food, germ-free fish did not exhibit myeloid cell accumulation in the intestine following sHCD feeding (Fig. 3d). As colonization with microbes contributes to intestinal development and function^32^, we ruled out the possibility of this affecting food uptake and transit in germ-free fish (Supplementary Figs 4d and 5d). In addition, equal uptake of cholesterol by intestinal epithelial cells was demonstrated using BODIPY-cholesterol by confocal analysis in conventionally or germ-free-raised fish (Supplementary Fig. 6). From these data we concluded that the accumulation of myeloid cells in the intestine mediated by cholesterol requires the presence of the commensal microbiota. However, in these experiments we could not exclude the possibility that cholesterol might also directly activate PRR^33^, leading to enhanced activation of signal 1. Therefore, we used *Tg(NFkB:EGFP)* fish (in which enhanced green fluorescent protein (EGFP) is expressed on NF-κB activation^29^) fed either ZM or HCD, to test for an enhanced activation of NF-κB following cholesterol feeding. Similar levels of EGFP were observed in HCD- and ZM-fed fish (Supplementary Fig. 7a), suggesting that HCD did not directly further activate the NF-κB pathway. In addition, by using *Tg(NFkB:EGFP)* crossed with *Tg(fms:mCherry)* fish we demonstrated that there was no statistically significant increase in the number of EGFP+/mCherry+ double-positive cells or the intensity of EGFP expression in mCherry+ macrophages following HCD (Supplementary Fig. 7b). This suggests that NF-κB is activated in non-haematopoietic intestinal cells at steady state and is not further activated in either these or mCherry+ cell compartments by HCD (Supplementary Fig. 7c). The increase in mCherry+ single-positive cell numbers reflects the increase in macrophages following HCD (Supplementary Fig. 7c), but these do not display NF-κB activation (that is, EGFP expression).

It has previously been shown that diets rich in fats/cholesterol can alter the microbiome^3^. We found no detectable change in the microbiota in the intestine after a single HCD meal, as no difference in the ratio of 16S:18S ribosomal RNA in either larvae or adults was observed (Supplementary Fig. 8a) subsequent to the HCD feed. Furthermore, analysis of the microbiome showed no statistically significant alterations in bacterial species composition, at any taxonomic level (Phylum down to operational taxonomic unit, analysed using White’s non-parametric *t*-test with correction for multiple testing, Benjamini–Hochberg false discovery rate), consequent to HCD feeding in larvae in terms of total transcript expression (Supplementary Fig. 8b). In addition, the weighted UNIFRAC distances between the HCD and ZM groups were not significantly different either. Overall analysis of the diversity showed no statistical difference between ZM- or HCD-fed groups (Supplementary Fig. 8c).

It is noteworthy that Kanther *et al*.^29^ demonstrated that the commensal microbiota stimulates constitutive NF-κB activation in intestinal epithelial cells, as EGFP expression and microbial colonization occurred simultaneously in dsRed-expressing epithelial cells in the intestine of *Tg(NFkB:EGFP)xTg(ifabp:dsRed)* fish. In summary, we conclude that HCD-induced intestinal immune cell accumulation is dependent on constitutive activation of signal 1 provided by the microbiota and that this aspect of inflammasome activation is not altered by cholesterol.

### HCD-induced inflammation is dependent on the inflammasome

Cholesterol uptake into intestinal epithelial cells via NPC1L1 might therefore provide signal 2, leading to activation of the inflammasome in epithelial cells. To test this hypothesis, we targeted several components of the inflammasome (Fig. 3a). In larval fish, we demonstrated that treatment with the NADPH oxidase and reactive oxygen species production inhibitor, VAS-2870, abrogated the HCD-induced intestinal accumulation of L-Plastin+ cells (Fig. 3e) in a dose-dependent manner (Supplementary Fig. 9a). Similar data were obtained using the Cathepsin B inhibitor, Ca-074-Me (Fig. 3f and Supplementary Fig. 9b). Caspase-1 (Caspase a and b in zebrafish) was also involved, as the caspase inhibitor, N-acetyl WEHD-al, inhibited the inflammatory cell infiltrate in larval zebrafish receiving HCD (Fig. 3g).

**Figure 4:**
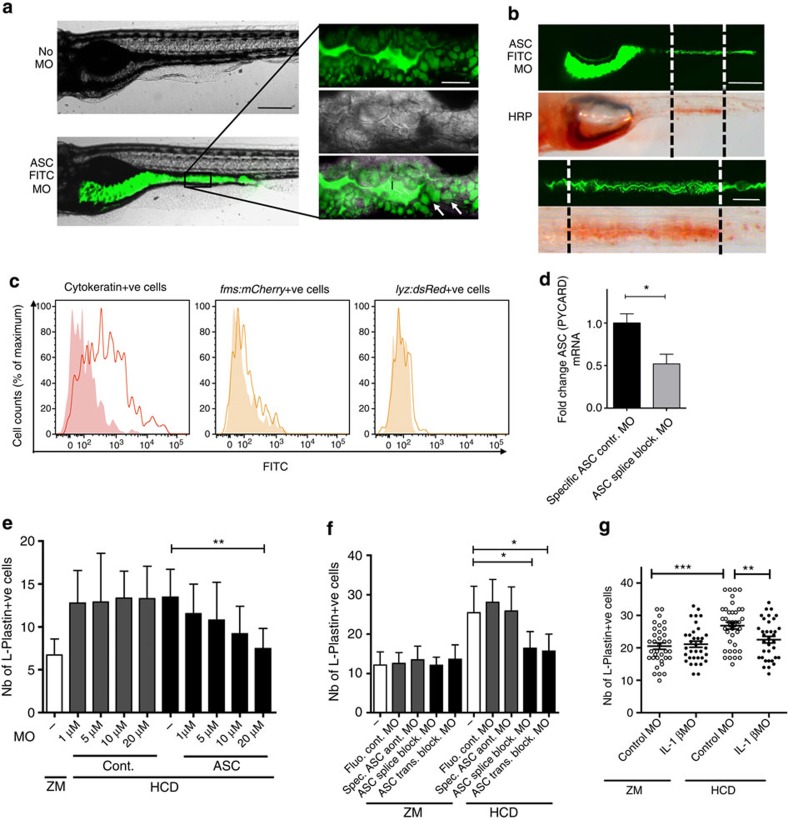
Treatment with ASC MO using a novel mode of delivery abrogates HCD-induced intestinal myeloid cell accumulation. (**a**) Representative fluorescent image of larvae treated with 20 μM FITC-conjugated ASC splice-blocking MO (FITC ASC MO, bottom panel) or left untreated (top panel). Insets highlight green fluorescence of FITC ASC MO in intestinal lumen (l) and in epithelial cells (white arrow). Scale bars, 100 μm (black); 20 μm (white). (**b**) Representative fluorescent and bright-field images of larvae treated with 20 μM FITC ASC MO (top panel) subsequently treated with 10 mg ml^*−*1^ HRP for 2 h. HRP was detected histochemically (bottom panel) and found to be localized in the same region as the MO (indicated by black and white dotted lines). Insets show enlarged images of the region containing specialized enterocytes that have taken up FITC ASC MO and HRP. Scale bars, 100 μm (top); 50 μm (bottom). (**c**) Histograms depict FITC fluorescence in intestinal cells of larvae treated with 20 μM FITC ASC MO (solid line) or untreated (filled histogram) and double-stained for cytokeratin (left plot), gated on dsRed+ cells in *Tg(lyz:dsRed)* (middle) or mCherry+ cells in *Tg(fms:mCherry)* (right). Pool of *n*≥10 larval intestines, representative of ≥2 experiments. (**d**) qRT–PCR analysis of FITC+ sorted intestinal cells of zebrafish larvae treated with either 20 μM of ASC-specific control MO or FITC ASC MO (pool of *n*=50–100 larval intestines per sample). Relative expression values were normalized to 18S and expressed as fold change relative to the control sample. Mean+s.e.m. of triplicates. Representative of two experiments. Mann–Whitney test. (**e**) Effect of increasing ASC or control MO concentration treatment on the total number of intestinal L-plastin+ cells. (**f**) Effect of different ASC MOs on the total number of intestinal L-plastin+ cells. (**e**,**f**) *n*≥15, one exact experimental replicate and more than three experimental replicates for the highest concentration of MO. Mean+95% confidence intervals. Kruskal–Wallis test. (**g**) Effect of IL-1β MO treatment the total number of intestinal L-plastin+ cells. *n*≥35, pooled from two experiments. Error bars: s.e.m. One-way analysis of variance. ***P<0.001, **P<0.01 and *P<0.05.

To assess the involvement of ASC (PYCARD), a central component of inflammasomes (Fig. 3a), we used a MO-based knockdown approach. As the knockdown efficiency of MO injected into single-cell stage embryos diminishes after 5 days post fertilization (dpf), especially in cells with a high renewal activity such as the intestinal epithelium, we devised a novel delivery method by simply adding the MO to the fish water 24 h before and during the 6-h feeding period (see Supplementary Fig. 1 for protocol). First, we validated this new method by imaging MO uptake in the intestine using a fluorescein isothiocyanate (FITC)-conjugated ASC splice-blocking MO and detected successful uptake in the epithelium (Fig. 4a). The uptake was seen exclusively within a defined region of the mid intestine previously described to contain specialized enterocytes that internalize luminal contents through pinocytosis, which can also be visualized using horseradish peroxidase (HRP)^34^ (Fig. 4b). Second, analysis by flow cytometry following treatment with the FITC-conjugated ASC splice-blocking MO revealed an increase in FITC fluorescence in Cytokeratin+ epithelial cells but not in mCherry+ macrophages in *Tg(fms:mCherry)*, or in dsRed+ neutrophils in *Tg(lyz:dsRed)* larvae (Fig. 4c), indicating MO uptake by intestinal epithelial but not by myeloid cells. Finally, we confirmed the ability of this MO to significantly knock down ASC mRNA by performing reverse-transcription PCR (RT–PCR) on injected embryos (Supplementary Fig. 10) and quantitative RT–PCR (qRT–PCR) on sorted FITC+ cells extracted from the intestines of larvae treated with either FITC-conjugated ASC-specific control MO or the FITC-conjugated ASC splice-blocking MO (Fig. 4d). For further proof-of-principle experiments to test for efficient knockdown, we used a GFP MO (not tagged with FITC) to target GFP in *Tg(ubi:EGFP*) fish^35^ and found significantly decreased fluorescence intensity in intestinal cells (Supplementary Fig. 11).

Both translation and splice-blocking MOs targeting ASC prevented the accumulation of HCD-induced L-Plastin+ cells in a dose-dependent manner (Fig. 4e,f) in larval fish, while none of the control MOs had any effect on the accumulation of myeloid cells mediated by HCD. Using food supplemented with fluorescently labelled microspheres, we showed that neither control nor ASC MO affected food uptake or transit (Supplementary Figs 4e and 5e). Together, these experiments demonstrated that we could efficiently target the ASC gene in the intestinal epithelium by addition of MO directly to the water, and that ASC, an essential component of the inflammasome, is involved in the accumulation of myeloid cells mediated by HCD.

Although we found an increase in IL-1β transcripts in the intestine of mice following HCD, this was not detectable in either zebrafish larvae or adults (Supplementary Fig. 12). Nevertheless, we used our novel MO-delivery approach to target IL-1β using a previously validated MO and found that knockdown resulted in abrogation of the accumulation in L-Plastin^+^ cells in the intestine (Fig. 4g), suggesting that this cytokine is involved in HCD-induced intestinal inflammation.

### HCD activates the inflammasome in intestinal epithelial cells

**Figure 5:**
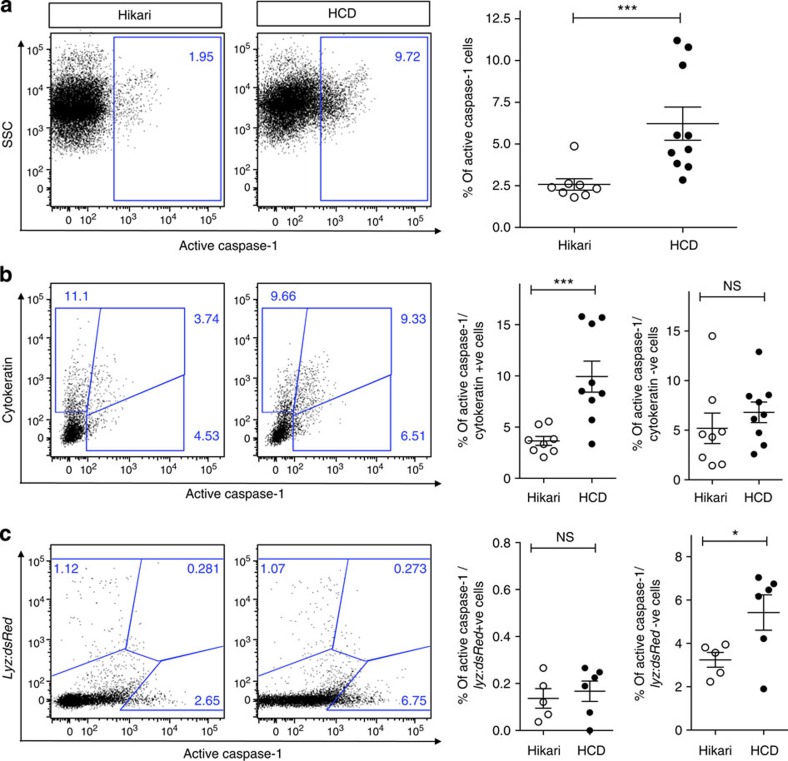
HCD-induced myeloid cell accumulation is dependent on inflammasome activation in intestinal epithelial cells. (**a**) Representative flow cytometry plots and quantification of intestinal tissue from adult zebrafish fed control (Hikari) or HCD for 6 h and treated with the active Caspase-1 FLICA substrate. *n*≥8. (**b**) Intestinal cells were fixed and double-stained with a cytokeratin antibody to label intestinal epithelial cells. *n*≥8. (**c**) Intestinal cells from *Tg(lyz:dsRed)* adult zebrafish were analysed following HCD and staining with FLICA. *n*≥5. In all graphs, each dot represents one adult, pooled from two experiments or more. NS, nonsignificant, Error bars are s.e.m. Mann–Whitney test. ***P<0.001 and *P<0.05.

We demonstrated inflammasome activation, directly triggered by cholesterol, in the intestinal epithelial cells by staining intestinal cells of adult zebrafish with an active Caspase-1-specific substrate, fluorescent-labelled inhibitor of caspase activity (FLICA). We found an increased percentage of active Caspase-1 (FLICA)-positive intestinal cells in HCD in comparison with Hikari-fed control fish (Fig. 5a). Co-staining with a cytokeratin antibody demonstrated the direct cholesterol-induced activation of Caspase-1 in epithelial cells (Fig. 5b), with a significant increase in the percentage of cells positive for both cytokeratin and FLICA following HCD, while there was no significant difference in these percentages in cells negative for cytokeratin. As it has previously been shown that the inflammasome could be activated by cholesterol in cultured macrophages^12^, it was important to assess whether myeloid cells could also play a role in our study. We used the *Tg(lyz:dsRed)* line, as this was the only transgenic available in our lab that expressed a fluorophore into adulthood, but did not exhibit a spectral overlap with the FLICA substrate. There was no change in the percentage of cells positive for both dsRed (*lyz* myeloid cells) and FLICA following HCD and the significant increase in FLICA-active Caspase-1-positive cells was clearly in the dsRed-negative population (Fig. 5c), that is, in the non-myeloid cells. These data together with the fact that ASC FITC MO could not be detected in these cells (Fig. 4c) strongly indicate that activation of inflammasome in resident myeloid cells is not necessary for the accumulation of myeloid cells in the intestine, following the acute feeding of HCD reinforcing the central role of inflammasome activation in intestinal epithelial cell in cholesterol-induced intestinal inflammation.

### Extended HCD impairs intestinal motility

**Figure 6:**
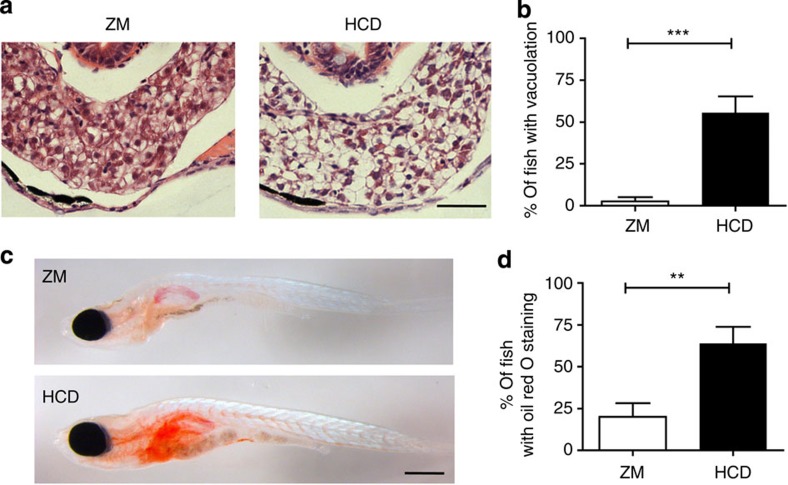
Extended HCD induces steatosis in the liver. (**a**) Representative images of paraffin transverse sections stained with haematoxylin and eosin of the liver of 15 dpf larvae fed ZM control or HCD for 10 days. Scale bar, 100 μm. (**b**) Percentage of fish showing vacuolation in the liver. *n*≥34, pooled from two experimental replicates. (**c**) Representative image of whole-mount Oil Red O-stained 15 dpf larvae fed ZM control or HCD for 10 days. Scale bar, 500 μm. (**d**) Percentage of fish showing Oil Red O staining in the liver. *n*=25, pooled from two experimental replicates. Error bars represent s.e.m. Mann–Whitney test. ***P<0.001 and **P<0.01.

**Figure 7:**
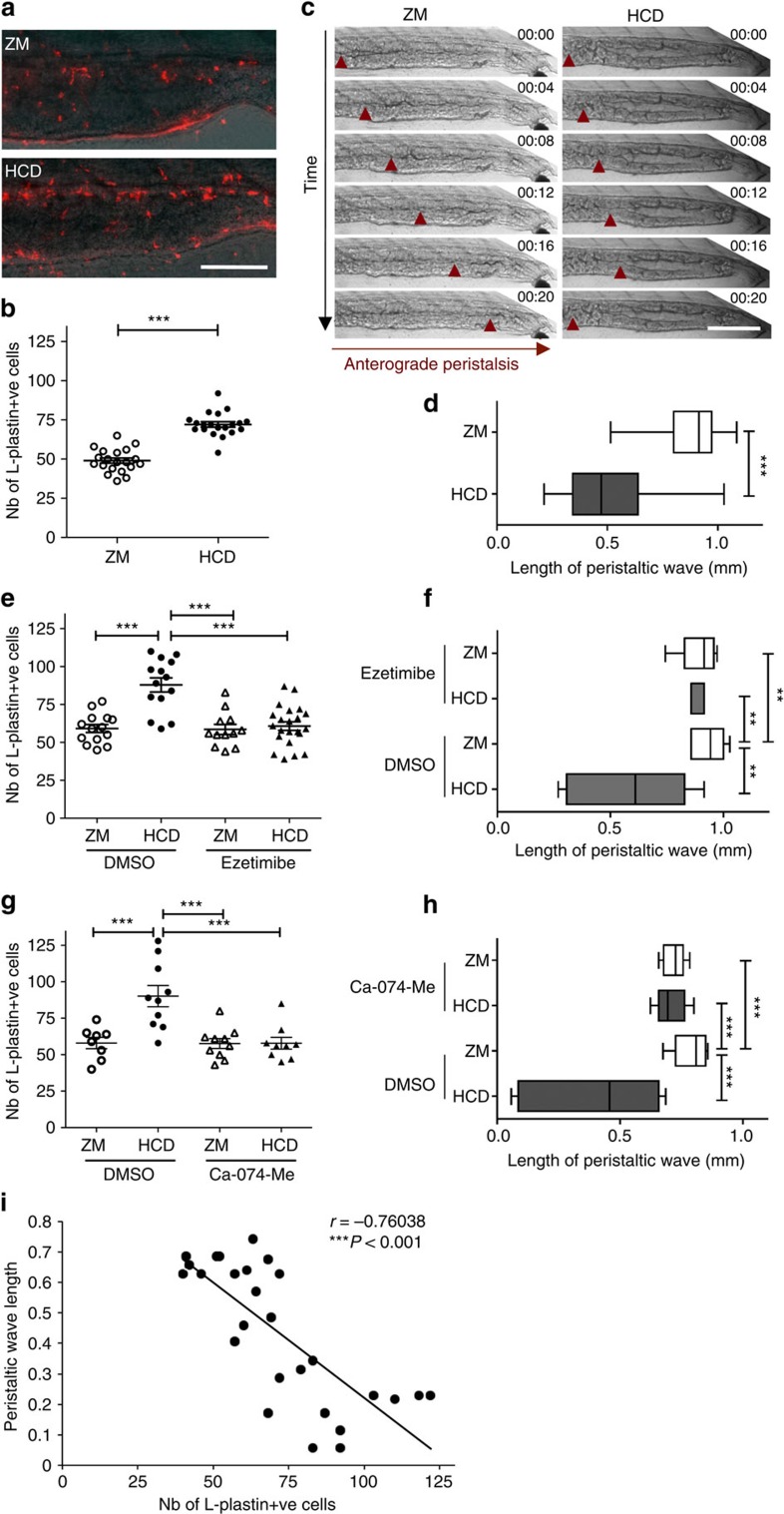
Extended HCD induces sustained inflammation and impaired peristalsis. (**a**) Representative images and (**b**) quantification of the total number of intestinal L-plastin+ cells of 15 dpf *Tra*^*−/−*^*/Nac*^*−/−*^ larvae fed HCD or ZM control for 10 days. *n*=20, one representative experiment of four. Scale bar, 150 μm. Two-tailed *t*-test. (**c**) Representative images and (**d**) average length of anterograde peristalsis with contractions (red arrowhead) over time (20 s) of the distal intestine of larvae fed (*n*=18). One representative experiment of three. Scale bar, 200 μm. Mann–Whitney test. (**e**) Total number of intestinal L-plastin+ cells (*n*≥12) and (**f**) average length of peristaltic wave (*n*≥6) of ezetimibe (25 μM)-treated larvae. One-way analysis of variance (ANOVA). (**g**) Total number of intestinal L-plastin+ cells (*n*≥8) and (**h**) average length of peristaltic wave (*n*≥10) of Cathepsin B inhibitor (Ca-074-Me, 10 μM)-treated larvae. One-way ANOVA. In **e**–**h**, one experiment of two is shown. (**i**) Correlation of peristaltic wavelength with the total number of L-Plastin+ cells in intestine. Pearson’s correlation coefficient: −0.76. Data are pooled from two experiments. ***P<0.001 and **P<0.01. Error bars represent s.e.m.

To explore the relevance of the zebrafish model for diseases associated with prolonged high-cholesterol exposure, larvae were fed HCD for 10 days (from 6 to 15 dpf; forprotocol, see Supplementary Fig. 13a). As anticipated^36^, cholesterol deposits were found in the caudal vein in HCD-fed fish (Supplementary Fig. 13b). We performed histological analysis of the liver and discovered evidence of steatosis (vacuolation) in 56% of HCD-fed fish (Fig. 6a,b) and lipid accumulation confirmed by Oil Red O staining (Fig. 6c,d). Further, we found a sustained intestinal accumulation of L-Plastin+ cells (Fig. 7a,b) in HCD-fed fish. Disturbance of intestinal motility without an identified origin is a hallmark of functional gastrointestinal disorders such as irritable bowel syndrome^37^. Further, patients recovering from abdominal surgery often suffer from postoperative paralytic ileus, suggesting a link between inflammatory responses in the intestine and impairment of gastrointestinal motility^38^,^39^,^40^. We therefore performed an analysis of gut motility by observing peristalsis after 10 days treatment with HCD. We observed a marked increase in the percentage of fish with incomplete peristalsis together with reduced average length of peristaltic waves (Fig. 7c,d and Supplementary Movie 1 and 2). Ezetimibe treatment showed that both sustained accumulation of L-Plastin+ cells and impaired peristalsis were dependent on cholesterol binding/uptake (Fig. 7e,f). Using the Cathepsin B inhibitor (Ca-074-Me), we observed that both number of L-Plastin+ cells and impaired peristalsis were dependent on inflammasome activation (Fig. 7g,h). Analysis revealed a striking, direct correlation between the number of L-Plastin+ cells present in the intestine and the level of impairment in peristalsis (Fig. 7i). Our data not only corroborates the notion of inflammation-induced functional intestinal disorder, but also shows that dietary components could contribute to these conditions. Overall, our results suggest that the acute inflammatory responses within the intestinal epithelium could be involved in well-characterized, long-term pathologies of the intestine.

## Discussion

Duewell *et al*.^12^ first described the ability of cholesterol crystals to induce inflammasome activation in cultured murine macrophages. Here we extend our understanding of the pro-inflammatory properties of cholesterol using a model that delivers cholesterol through its normal route of ingestion to the intestinal epithelial cells of the whole organism. The concept of nutrient-induced acute intestinal inflammation has arisen from *in vitro* experiments in which fatty acids modulate cytokine production in intraepithelial lymphocytes^5^ and intestinal epithelial cells^6^. Any resulting local inflammation could explain why and how HFD compromises the intestinal integrity resulting in a leaky barrier. Such leaky barriers have been suggested as the mechanism by which intestinal bacterial products can translocate across the epithelium and induce local intestinal as well as systemic inflammation. However, there are no reports showing that nutrients can induce activation of the intestinal epithelial cells in an intact organism, which then leads to local inflammation. *In vitro* experiments using epithelial cells are hampered by the difficulty of delivering water-insoluble compounds such as cholesterol in a manner that mimics that of dietary uptake. In this study we have demonstrated a direct pro-inflammatory effect of ingested cholesterol through inflammasome activation in the intestinal epithelium of intact zebrafish, uncovering a novel route for the initiation of intestinal inflammation by dietary compounds.

Components of the inflammasome expressed in haematopoietic and in intestinal epithelial cells have been shown to play a critical role in maintaining intestinal homeostasis and sensing the microbiota, as well as in regulating tissue repair and regeneration through orchestrating mucosal immunity^7^,^8^. However, the roles of these components in models of human intestinal inflammatory diseases such as IBD remain controversial. Indeed, conflicting results have been reported using the dextran sulphate sodium (DSS)-induced intestinal autoinflammation model. Although mice deficient in Caspase-1 or NLRP3 showed decreased severity of intestinal DSS-induced pathology, in other studies mice deficient in Caspase-1, NLRP3, 6 or ASC exhibited increased disease severity (reviewed in Strowig *et al*.^41^). The difference in these outcomes not only emphasizes the complex nature of inflammasome activity in mucosal responses, but also suggests the possibility of different inflammasome influence (that is, beneficial versus detrimental) within different cellular compartments (that is, haematopoietic versus intestinal epithelial cells). Indeed, there is growing evidence for the importance of NLRP and other inflammasome components in non-haematopoietic cells of the intestine. The use of bone-marrow chimeras showed that in infections with pathogenic *Citrobacter rodentium*, protection and intestinal inflammation were dependent, respectively, on the expression of NLRC4 or ASC and NLRP3 in non-haematopoietic cells^42^,^43^. Further, NLRP12 deficiency in non-haematopoietic intestinal cells was in part responsible for increased susceptibility to DSS-induced colitis, especially in later phases of the disease^44^. Overall, these studies suggest an important role of inflammasome components in epithelial cells in the initiation of inflammation and tissue repair, and also highlight the need for novel tools, such as conditional knockouts, to study the involvement of cell-type-specific inflammasome expression in inflammatory disease processes.

In our model of HCD-induced intestinal inflammation, inflammasome activation by cholesterol was not found in haematopoietic cells. Our experiments showed that although we were able to demonstrate the involvement of Caspase-1-like molecules (Caspase a and b in zebrafish) using FLICA and ASC/Pycard MO in intestinal epithelial cells, we found no evidence of their involvement in myeloid cells investigated, that is, dsRed+ cells in *Tg(lyz:dsRed)* fish. Further, our finding that targeting inflammasome components such as ASC using this MO delivery technique abrogated myeloid cell accumulation following HCD confirms the previously proposed relevance of these specialized enterocytes in intestinal immune responses^34^. Indeed, Wallace *et al*.^34^ suggested that this is the region of the small intestine that may contain cells with antigen-presenting capacity. Genome analysis in zebrafish has identified six genes in the NLR-B subfamily of NLR genes that correspond to the NALPs in mammals but with, as yet, no clearly defined orthologues^45^, and therefore we did not make an attempt to characterize which NLR gene(s) was involved here. However, when this gene family is further characterized, our new MO delivery method in zebrafish will offer the possibility to selectively knock down each member of the gene family in the specialized enterocytes of the intestinal epithelium, thereby identifying their role.

Although the inflammasome is essential in sensing danger and triggering immune and repair machinery, our study demonstrates that dietary components such as cholesterol can also be detected by the epithelial cells and activate the inflammasome, leading to accumulation of various myeloid cells in the intestine. The magnitude of accumulation of myeloid cells found in the intestine was interestingly within the same range as that seen in a model of DSS-induced intestinal inflammation in zebrafish^46^. However, although pro-inflammatory cytokine transcripts were detected in the zebrafish DSS model this was not the case in our zebrafish model, although an increase in IL-1β mRNA was detected in the mouse ileum following cholesterol feeding. Nevertheless, IL-1β knockdown by MO abrogated myeloid cell accumulation in the intestine following HCD, showing that this pro-inflammatory cytokine is involved in this local inflammatory response. These results may reflect the possibility that the inflammation triggered by a single meal high in cholesterol induces only low levels of IL-1β expression, which are undetectable by qRT–PCR and, perhaps not surprisingly, minimal when compared with those induced by broad chemical damage by agents such as DSS or trinitrobenzenesulfonic acids. Importantly, prolonged exposure to HCD did have deleterious functional effects in the intestine with impaired peristalsis that correlated with myeloid cell accumulation, characteristic of intestinal inflammatory disorders^37^. Whether our model of HCD-induced acute and chronic intestinal inflammation resembles human IBD in the long-term remains to be fully assessed, but it may offer a physiological and relevant model for studying aspects of intestinal inflammation. Further, this model provides routes to reduction and refinement in animal experimentation, as some aspects of intestinal pathophysiology of zebrafish larvae can be visualized using longitudinal and non-invasive imaging techniques.

We demonstrated that functional impairment of the intestine and inflammation following prolonged HCD was dependent on inflammasome activity and cholesterol binding/uptake, as both an inhibitor of Cathepsin B and cholesterol uptake abrogated this impairment. These data suggest that responses induced by dietary components, such as cholesterol, may be involved in triggering intestinal inflammatory disorders, such as IBD, or exacerbating disorders of other aetiology. Although there is growing interest in diet-mediated alterations in the microbiota^3^,^4^ and the possibility that such changes are related to disease aggravation, our results show that primary and direct inflammatory effects of dietary components on the intestinal epithelium should now also be taken into account.

It is clear from clinical studies that potentially life-threatening diseases such as obesity, cardiovascular disease, type 2 diabetes, metabolic syndrome, colorectal cancer and IBD are associated with prolonged intake of Western-type diets rich in high fat/cholesterol, high protein and high sugar^1^,^2^. Despite their multifactorial nature, the induction of inflammation is a pathophysiological response common to these chronic, often systemic, autoinflammatory conditions^47^ and this association is underpinned mechanistically by evidence of coordinated regulation and shared usage of signalling molecules and pathways between lipid metabolism and inflammatory responses^48^, both of which are evolutionarily conserved between species, including the zebrafish^13^,^14^,^15^. Here, extended HCD, beyond functional impairments in the intestine, induced hepatic steatosis and vascular lipid accumulation, the latter of which, as previously reported by Stoletov *et al*.^36^, is accompanied by vascular inflammation. As cholesterol is first encountered, absorbed and metabolized by the intestinal mucosa and elicits an inflammatory response in intestinal epithelial cells, which is involved in long-term local pathologies, we believe it is of importance to further investigate the impact of this local inflammation on systemic conditions. Recent evidence has emerged, suggesting that intestinal inflammation precedes and therefore is associated with diet-induced systemic conditions^4^, and further that cholesterol absorption from the gut has been shown to play a role in most of these conditions^49^,^50^. However, its relevance for systemic inflammation in metabolically relevant tissues is controversial^49^ and will remain the subject of future investigations.

In conclusion, our study reveals direct pro-inflammatory effect of ingested cholesterol through inflammasome activation in the intestinal epithelium, leading to inflammation in the intestine. Inflammasome activation resulted in an accumulation of inflammatory leukocytes around the intestine and in the longer term, still consequent to cholesterol binding, local and systemic pathologies. This model, demonstrating clearly the inflammatory effects of dietary components, extends our knowledge of how dietary choices can influence processes involved in chronic inflammatory disorders.

## Methods

### Zebrafish and mouse care and breeding

Fish and mice were maintained according to standard practices and all procedures conformed to the UK Home Office regulations (ASPA 1986). Female Balb/c mice (8–10 weeks old; Charles River, UK) were maintained on a 12 h light/12 h dark cycle. Fish were reared and maintained at 28.5 °C on a 14 h light/10 h dark cycle. The respective project licenses are PPL 70/7472 and 70/6963. The transgenic zebrafish lines *Tg(pu.1:GFP)*^24^, *Tg(mpx:GFP)* (*Tg(mpx:GFP)i114*)^26^, *Tg(ubi:EGFP)*^35^, *Tg(lyz:dsRed)*^20^, *Tg(fms:mCherry) (Tg(fms:Gal4.VP16)i186;Tg(UAS:nfsB.mCherry)i149*)^25^, *Tg(NFkB:EGFP) (Tg(pNF-κB:EGFP)sh235)*^29^ and *Tra*^*−/−*^*;Nac*^*−/−*^ mutant (Tübingen) were used.

### Preparation of experimental diets

Cholesterol (C75209; Sigma) was dissolved in diethyl ether (Sigma) to create a 10% (or a 5%) solution, of which 400 μl was added to 0.5 g of standard zebrafish larval food (ZM; ZM Systems, ingredients: protein 52%, oil 12%, ash 8%, moisture 7%, fibre 3%) or adult food (Hikari, Hikari, ingredients: protein 49%, oil 7.8%, ash 11%, moisture>10%, fibre 0.9%, phosphorus 1.7%), to create cholesterol-enriched diets for acute and extended feeding, respectively. Four hundred microlitres of diethyl ether was added to 0.5 g of ZM or Hikari to serve as a control diet. The diets were left overnight for the ether to evaporate and ground up the following day into fine particles using a pestle and mortar (adapted from Stoletov *et al*.^36^). For the preparation of sterile food (sZM, sHCD) for GF experiments, ZM was autoclaved before the supplementation with cholesterol as described above, which was performed under sterile conditions.

### Feeding of zebrafish larvae and adults

Before experimental procedures, larvae were initially reared at 28.5 °C at a maximum density of 50 larvae per Petri dish in system water containing 3 × 10^*−*5^% methylene blue (Sigma) as an antifungal agent and 30 mg l^*−*1^ 1-phenyl 2-thiourea (Sigma) to prevent melanization (except for *Tra*^*−/−*^*;Nac*^*−/−*^ mutant). Great care was taken to remove unfertilized eggs and chorions post hatching. Zebrafish larvae (6 dpf) were placed in system water containing clotted cream (63.5 g of fat, 170 mg of cholesterol, 2.2 g of carbohydrate and 1.6 g of protein per 100 g) used at a 1:10 dilution, cholesterol, or a standard diet (ZM) for 6 h at 28.5 °C^18^. The number of neutrophils and macrophages was quantified at indicated time points after the 6-h-feeding period by counting cells (in a blinded manner) that were present in the intestine of live *Tg(mpx:GFP)*, *Tg(lyz:dsRed)* and *Tg(fms:mCherry)* transgenic zebrafish, respectively, whereas myeloid cells were quantified in the intestine of WT zebrafish after fixation and immunolabelling with an L-plastin antibody. Significant differences in myeloid cell accumulation were found using doses of ≥4% cholesterol with groups of about 30 larvae and power calculations determined significant differences for 2% cholesterol when using *n*=190 (Supplementary Fig. 2a) These concentrations of cholesterol correspond to doses given to genetically non-predisposed mice^27^. Adult zebrafish were starved for 48 h before feeding for 6 h with cholesterol or standard diet (Hikari). For extended feeding, zebrafish larvae were fed twice a day for 10 days with cholesterol-enriched or standard ZM diet (see Supplementary Fig. 13a for protocol). For experiments studying lipid deposition in the caudal vein, ZM and HCD were supplemented with 10 μl ml^*−*1^ of a fluorescent lipid probe, cholesteryl BODIPY 576/589 C11 (Invitrogen) according to Stoletov *et al*.^36^ For all experiments involving feeding, zebrafish larvae were randomly assigned to the various treatment groups. After randomized allocation of animals, treatments were coded until the experiment was finished and data were analysed. A sample size of about 15 zebrafish larvae per treatment group in which the readout was quantification of intestinal inflammatory cells was calculated based on the magnitude ±s.d. (time zero before feeding: 8.3±4.1; 18 h post ZM feeding: 11.09±5.05; 18 h post HCD feeding: 19.57±9.11) identified in a pilot study and was used for all further experiments involving feeding.

### Feeding of mice and tissue harvest

Age- and gender-matched female Balb/c mice (8–10 weeks old; group of 3) were starved for 12 h during the light phase before the oral gavage at the beginning of the dark phase with 200 μl of either melted butter (unsalted, 82.2 g of fat and 213 mg of cholesterol per 100 g), corn oil, corn oil with cholesterol (40 mg ml^*−*1^) or water control. The intestinal tract was dissected after 12 h for flow cytometry and RNA extraction (see details for qRT–PCR below). The intestine was flushed twice with PBS, the Peyer’s patches removed and the intestine separated in three sections: upper (containing the duodenum and the proximal jejunum) and lower (containing distal jejunum and ileum) parts of the small intestine and the colon. Tissue samples from the ileum were preserved in RNAlater (Ambion) for RNA extraction. For flow cytometry, tissue was cut into small pieces (1 cm) and processed immediately.

### Flow cytometry of disaggregated mouse intestinal tissue

Tissue was incubated in PBS supplemented with 10 mM EDTA and 10% FCS for 10 min at 37 °C. This step was repeated four times, followed by incubation in RPMI containing 10% FCS, 15 mM Hepes, 100 U ml^*−*1^ collagenase VIII and 50 U ml^*−*1^ DNAse for 45 min at 37 °C. The suspension was filtered (100 μm) and cells separated using Percoll. For flow cytometry, cells were blocked in with 0.5 μg ml^*−*1^ anti-FCγRII/FCγRIII antibody (553141; BD) for 20 min at 4 °C, followed by an incubation with a CD11b-FITC (1:100; 553310; BD) or a CD11c-FITC (1:100; 557400; BD) antibody in PBS containing 3% BSA and 0.09% sodium azide. Samples were processed on a LSRFortessa (BD) and analysed using FlowJo software (Tree Star).

### L-plastin antibody staining of zebrafish larvae

Following fixation, larvae were washed in PBS and then in dH_2_O. If counterstained with a HRP secondary antibody, larvae were incubated in 0.5% peroxidase solution (0.5% hydrogen peroxide (H_2_O_2_) in PBS) for 20 min, to block endogenous peroxidase activity. They were then permeabilized in acetone for 7 min at −20 °C, rinsed in dH_2_O and then incubated in blocking solution (5% donkey serum, 1% dimethylsulphoxide (DMSO) and 0.1% Tween in PBS) for 30 min. Larvae were incubated overnight at 4 °C in rabbit anti-zebrafish L-plastin antibody (kindly provided by Paul Martin, University of Bristol, UK; diluted 1:500 in blocking solution), after which they were washed three times for 20 min in PBST and then twice for 20 min in blocking solution. Larvae were then incubated either for 4 h in secondary donkey anti-rabbit IgG conjugated to TRITC (711-025-152; Jackson ImmunoResearch Laboratories; 1:100 diluted in blocking solution) or in secondary goat anti-rabbit IgG conjugated to HRP (111-035-003; Jackson Immunoresearch) at 4 μg ml^*−*1^ in blocking solution. In the case of the HRP antibody, fish were incubated for 30 min in 0.3 mg ml^*−*1^ diaminobenzidine in PBS, followed by incubation for 10 min in 0.3 mg ml^*−*1^ diaminobenzidine and 0.05% H_2_O_2_ in PBS, and a two final washing steps in PBST. Larvae were then imaged using an epifluorescent microscope and total cell numbers were counted in a blinded manner within the larval intestine.

### Flow cytometry of disaggregated zebrafish intestinal material

For analysis of cell recruitment, distal portions of adult zebrafish intestines were dissected, dissociated into single-cell suspensions by digestion for 15 min at 37 °C with PBS containing 0.25% trypsin, 1 mM EDTA and 5 mg ml^*−*1^ collagenase P (Roche), and subjected to flow cytometry. For analysis of neutrophils, cells from *Tg(mpx:GFP)* transgenics were analysed as live cells, whereas for analysis of myeloid cells, dissociated cells were fixed for 30 min at room temperature in 4% paraformaldehyde (PFA) and stained for 1 h at room temperature with an rabbit anti-zebrafish L-plastin antibody (diluted 1:500 in blocking solution containing 5% donkey serum in PBS), then for 30 min at room temperature with the secondary donkey anti-rabbit IgG conjugated to TRITC (diluted 1:500 in blocking solution) and then analysed by flow cytometry. For analysis of fluorescence intensity and EGFP+ cell number, intestines of larval *Tg(NFkB:EGFP)* transgenic zebrafish were dissected out, pooled (*n*=3), filtered through a 100-μm strainer and analysed by flow cytometry. For analysis of Caspase-1 activation, distal portions of adult zebrafish intestines were strained through a 100-μm strainer to prepare single-cell suspensions, which were then incubated for 30 min at 28.5 °C in 1 × FLICA using the FAM FLICA Caspase-1 Assay Kit (ImmunoChemistry Technologies) according to the manufacturer’s instructions, washed in wash buffer and analysed by flow cytometry in the presence of 1 μg ml^*−*1^ DAPI (Sigma) for live/dead cell discrimination. For co-labelling with cytokeratin, single-cell suspensions were fixed after incubation with FLICA, according to the manufacturer’s instructions, washed twice in PBS–Triton X (0.1%), stained for 1 h at room temperature with a mouse anti-pan-cytokeratin antibody (MA1-82041; Thermo Scientific, diluted 1:100 in blocking solution containing 5% goat serum in PBS) and then washed and stained for 30 min at room temperature with the secondary goat anti-mouse-IgG-AF633 (A21052; Life Technologies; 1:1,000 in blocking solution). For analysis and sorting of FITC+ intestinal cells following treatment with indicated FITC-conjugated MOs, pools of dissected intestines were strained through a 100-μm strainer to prepare single-cell suspensions and were analysed in the presence of 1 μg ml^*−*1^ DAPI (Sigma) or fixed and co-labelled with a cytokeratin antibody as described above. All samples were processed using a LSRFortessa (BD) and analysed using FlowJo (Tree Star) or sorted using a FACS Aria (BD).

### qRT–PCR and RT–PCR

Dissected guts from larvae (pool of 20) or individual adults or from FACS-sorted FITC+ cells or from mouse intestinal tissues were homogenized using a pestle in lysis buffer and processed for RNA using the MagMAX-96 Total RNA Isolation Kit, according to the manufacturer’s instructions. The quantity and quality of RNA was assessed spectrophotometrically using a Thermo Scientific NanoDropTM 1000. Eighty-five nanograms of total RNA was used for reverse transcription using High-Capacity cDNA Archive Kit (Applied Biosystems), according to the manufacturer’s instructions. RT–PCR was performed to amplify unspliced and spliced ASC mRNA expression following injection with ASC splice blocking MO using the following primers Exon 1 Forward 5′- CGCGTCACAAAGTCTGCAAT -3′, Exon 2 Reverse 5′- CATCAGAGGGAGCACCTTTGC -3′ and Intron 1 Reverse 5′- CTCTGTCTGAATTTCCCGCCT -3′, and the Qiagen Taq PCR Master Mix Kit for 30 cycles at 62 °C annealing temperature. qRT–PCR was performed with 2% of complementary DNA generated using Taq fast universal 2 × PCR Master Mix (Applied Biosystems) and Taqman primer and probes assays for 18S, mouse *Il1β* (Mm01336189_m1), *Il18* (Mm00434225_m1), *Nlrp3* (mm00840904_m1), *Nlrc4* (Mm01233151_m1), *Nlrp6* (Mm00460229_m1), zebrafish ASC *(pycard*, Dr03125114_m1, exon spanning 1-2, probe sequence: 5′- TGAGGAACACAGGGCAATCAGAAAG -3′), and zebrafish *il1β* (Dr03114368_m1, Applied Biosystems) and Sybr Green fast universal 2 × PCR Master Mix (Applied Biosystems) for 16S with the following primers F: 5′ TCCTACGGGAGGCAGCAGT 3′ and R: 5′ GGACTACCAGGGTATCTAATCCTGTT 3′. All reactions were performed in duplicate using a 7500 Fast Real-time PCR system (Applied Biosystems). Cycle thresholds obtained were normalized to 18S and calibrated to control food-fed fish samples for relative quantification.

### Treatment with inhibitors

WT zebrafish larvae (5 dpf) were pretreated in 25 μM ezetimibe (Sequoia Research Ltd) overnight, followed by feeding the next day for 6 h. For all other inhibitors, WT larvae (6 dpf) were pretreated for 30 min in Caspase-1 and 5 inhibitor *N*-Acetyl WEHD-al (Sigma), Cathepsin B inhibitor Ca-074-Me (Calbiochem), NADPH oxidase inhibitor VAS-2870 (Enzo Life Sciences) and NF-κB-activation inhibitor NAI (Merck), followed by feeding for 6 h. The inhibitors were administered into the water and were present for the duration of the feeding. For extended feeding experiments, inhibitors were present for the duration of the experiment with daily media and drug changes. Fish were fed twice daily. Inhibitors were initially used at a range of concentrations to determine an optimum concentration, and then further experiments were carried out using the optimum concentration and with an appropriate vehicle control as indicated in the main text or figure legends.

### Treatment with MOs

MO oligonucleotides were purchased from GeneTools, LLC (Philomath, OR, USA): 5′- GGTGCTCCTTGAAAGATTCCGCCAT -3′ (zebrafish ASC translational blocking MO), 5′- CAATTGCACTTACATTGCCCTGTGT -3′ (ASC splice blocking MO), 5′- CAATTCCAGTTAGATTGCCGTGTCT -3′ (ASC specificity control for splice blocking), 5′- CCCACAAACTGCAAAATATCAGCTT -3′ (IL-1β splice blocking MO), 5′- CCTCTTACCTCAGTTACAATTTATA -3′ (standard control MO) and 5′- ACAGCTCCTCGCCCTTGCTCACCAT -3′ (GFP MO) tagged with 3′-carboxyfluorescein (FITC) or not and diluted in system water. Six-dpf zebrafish larvae were exposed to 20 μM MO solution for 24 h before feeding at 7 dpf (see Supplementary Fig. 1 for protocol). To assess the efficiency of this delivery method, larvae were first treated with FITC-conjugated control MO and imaged. Figure 4a shows that FITC localizes to the epithelium of the intestine of treated fish. Next, *Tg(ubi:EGFP)* zebrafish larvae, which express EGFP ubiquitously, were treated at 6 dpf with an MO targeting GFP and fluorescence intensity quantified in the intestine after 48 h. Although GFP protein is a stable protein with a long half-life, we reasoned that a successful temporary knockdown should lead to a decreased production and, therefore, a detectable decrease in fluorescence intensity. Supplementary Fig. 11a shows that GFP intensity in the intestine of larvae treated with GFP MO was reduced compared with those treated with control MO. In addition, cell suspensions prepared from the dissected intestine tissue showed reduced mean fluorescence intensity by flow cytometry (Supplementary Fig. 11b) in GFP MO-treated fish compared with controls. Altogether, these results indicate that delivering MO in the water in 6 dpf larvae for 48 h induced partial knockdown of GFP expression in the intestine.

### GF zebrafish

Zebrafish larvae were reared as GF, as described previously^51^, with a few modifications. Briefly, freshly fertilized zebrafish eggs were maintained at 28.5 °C in sterile E2 containing a mixture of antibiotics (100 μg ml^*−*1^ ampicillin, 5 μg ml^*−*1^ kanamycin and 250 ng ml^*−*1^ amphotericin B) until 6 hpf when they were washed in 0.1% polyvinylpyrrolidone–iodine complex, rinsed three times with the E2 antibiotic medium, incubated in 0.003% sodium hypochlorite for 20 min at room temperature and washed again three times with the E2 antibiotic medium. Subsequently, embryos were transferred into tissue culture flasks at a density of five embryos per 10 ml of sterile E2 antibiotic medium and raised at 28.5 °C with daily media changes, but without any food supplementation until 6 dpf. Sterility of embryos was monitored routinely by spotting 100 μl of embryo media or lysed embryos on tryptic soy agar plates and was further confirmed by qRT–PCR using primers targeting 16S ribosomal RNA genes. For each experiment, conventionally raised (CONV) zebrafish littermates were maintained using standard conditions as described above, in the absence of any antibiotics or sterilization by bleaching.

### Histology

Stained larvae were embedded in a 3% agar block, dehydrated and paraffin wax embedded. Four-micrometre transverse sections were cut using a rotary microtome (Leica) and mounted onto glass slides, dried, deparaffinized in xylol, rehydrated and counter-stained with haematoxylin and eosin. Stained sections were imaged using Axiovison Rel 4.7 software on a Leica MPS 60 microscope fitted with an Axiocam HRc camera.

### Analysis of food uptake and intestinal transit

According to the previously published technique for measuring intestinal motility and transit in zebrafish larvae^52^, HCD or sHCD were supplemented with non-metabolizable 2-μm yellow, green or red fluorescent polystyrene microspheres (Invitrogen, F8827 or F8826) and fed to zebrafish larvae in the presence of indicated inhibitors, MOs or under GF conditions. To assess the amount of ingested food at 2 h, thresholded binary patterns of the area of the anterior bulb filled with fluorescent microsphere-labelled HCD were quantified using ImageJ (Supplementary Fig. 4a). This novel strategy to estimate food intake in larval zebrafish was validated as a method able to detect differences in the amount eaten, as a high concentrations (200 nM) of the NF-κB activation inhibitor NAI significantly decreased the amount of food ingested by larvae when compared with those treated with DMSO control or NAI at a concentration of 150 nM (Supplementary Fig. 4c). In addition, the number of fish that have ingested food at 2 h and 6 h was recorded. To assess kinetics of microsphere movement through the intestine, the transit zones and scoring system established by Field *et al*.^52^ was followed (Supplementary Fig. 5a) and larvae were analysed directly after the feeding period (6 h) and after 24 h (6 h feeding+18 h).

### Cholesterol uptake assay using BODIPY-cholesterol

BODIPY-cholesterol (Avanti Polar Lipids, Alabaster, AL) solutions were prepared according to Walters *et al*.^16^, with few modifications. In brief, BODIPY-cholesterol was dissolved in sterile DMSO to obtain a 1.5-mg ml^*−*1^ stock solution, which was subsequently diluted in 1% fatty-acid-free BSA in either system water or sterile E2 antibiotic medium. This suspension was further diluted directly by addition to larvae in system water or sterile E2 antibiotic medium, to obtain a final concentration of 1.5 μg ml^*−*1^ BODIPY-cholesterol. Larvae were fed for 6 h with sterile HCD before the uptake of BODIPY-cholesterol in intestinal epithelial cells was analysed by live confocal microscopy.

### Microbiota

cDNA was used as described above and from this source material 16S rRNA gene amplicons were generated for the V3–V5 variable regions using primer pair 357F and 926R. The amplicons were prepared by Research and Testing Ltd (Austin, Texas, USA) and were run on a Roche 454 GS FLX+. Once the sequence data were obtained, it was pre-processed using the ribosomal database project pyrosequencing pipeline using the default filtering criteria (*Q* score>20, max number of *N*’s=0, >200 and <600 nt). Once the sequences had been processed, they were run through Mothur using the Schloss SOP^53^. In addition, the shared OTU data set was edited to compress all singletons and OTUs for which there were <10 reads in a sample, into a holding OTU, called OTU_X, to preserve the same read numbers for each sample. Heatmaps of shared OTU data were generated in R using the packages NMF, RColorBrewer and Ggplots. All gut microbiota were sampled to >97% coverage with reads between 603 and 1,965 per sample. Statistical analysis of the changes in the bacterial OTUs was undertaken in STAMP^54^ using Welch’s *t*-test and using a Bonferroni correction for multiple testing.

### Microscopy

For live imaging, zebrafish were anaesthetized in MS222 and positioned laterally. Widefield fluorescence microscopy on *Tg(NFkB:EGFP)* larvae was performed by recording a series of *Z*-plane images (every 10 μm) on a Zeiss Axiovert 200 microscope with Compix SimplePCI imaging software. Images were merged and the intestine area selected using the image software Volocity and blind counts were carried out to assess the number of GFP+ cells in the distal intestine. Fluorescence microscopy on all other fish was performed on an Olympus CKX41 fluorescent microscope (Olympus UR FLT50) using the Q-Capture Probe software. Image analysis was performed using ImageJ software. To determine the fluorescence intensity, the intestine area was selected and the area, mean grey value, integrated density and raw integrated density measured. Five regions of the image background were selected and the mean grey value measured. The mean of the mean grey values of the background is used to calculate the corrected fluorescence using the following equation:


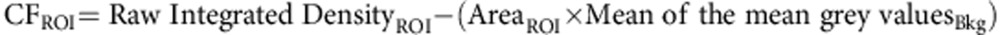


Bright-field imaging was performed to analyse intestine peristalsis. For that, intestinal motility was recorded at 1-s intervals for 2.5 or 5 min. The image sequence was converted into a movie using Volocity software, and waves of peristalsis measured and counted.

### MPO staining

Following fixation in 4% PFA (Sigma) overnight at 4 °C, the larvae were stained for MPO activity. Briefly, larvae were washed in PBST and then incubated with 0.075 mg ml^*−*1^ diaminobenzidine and 0.03% H_2_O_2_ in PBS for 2 h at 28.5 °C. Embryos were washed twice with PBS. Cell numbers were counted in a blinded manner.

### Oil Red O staining

Larvae were washed twice in PBS, incubated in 60% isopropanol for 1 h, followed by incubation with freshly prepared Oil Red O staining solution (0.3% Oil Red O in 60% isopropanol) for 2 h. After staining, samples were washed twice for 30 min in 60% isopropanol before being transferred to PBS.

### HRP staining

To assess HRP uptake into intestinal cells, 7-dpf larval zebrafish were incubated in system water containing 10 mg ml^*−*1^ HRP for 2 h at 28.5 °C. Following incubation, larvae were washed once in system water and fixed in 4% PFA for 2 h at room temperature. Following fixation, larvae were washed twice in PBS, twice in dH_2_O, transferred to acetone for 7 min at −20 °C, rinsed in dH_2_O and incubated at room temperature for 10 min with the AEC Peroxidase Substrate Kit (Vector Laboratories).

### Statistical analysis

All statistical analysis was carried out using GraphPad Prism 4.0 software (GraphPad Software, CA, USA). Normality distribution was tested with the D'Agostino–Pearson omnibus test. When comparing two groups, unpaired two-tailed *t*-tests (followed by Welch’s correction test for non-equal s.d.) and Mann–Whitney tests were used for parametric and non-parametric data sets, respectively. When comparing more than two groups, one-way analysis of variance followed by Tukey’s or Bonferroni’s multiple comparison test and Kruskal–Wallis test followed by a Dunn’s multiple comparison test were used for parametric and non-parametric data sets, respectively. Two-way analysis of variance followed by Bonferroni’s multiple comparison test was used to compare time-course curves. *P*-values of <0.05 were deemed statistically significant with ***P<0.001, **P<0.01 and *P<0.05. Pearson’s correlation coefficient was used to assess the significance of the correlation shown in Fig. 7i.

## Author contributions

F.P., N.J.S., J.S., J.R.L., L.B. and M.J.D. designed and interpreted experiments. F.P., N.J.S., N.Y., M.M.B, J.C., A.S. and L.B. performed experiments and analysed data. J.R.M. performed the microbiome analysis. F.P., J.R.L, L.B. and M.J.D. wrote the manuscript.

## Additional information

**How to cite this article:** Progatzky, F. *et al*. Dietary cholesterol directly induces acute inflammasome-dependent intestinal inflammation. *Nat. Commun.* 5:5864 doi: 10.1038/ncomms6864 (2014).

## Supplementary Material

Supplementary Figures Supplementary Figures 1-13.

Supplementary Movie 1 Representative movie of anterograde peristalsis in the distal intestine of 15 dpf Tra-/-/Nac-/- larvae fed ZM control diet for 10 days.

Supplementary Movie 2 Representative movie of anterograde peristalsis in the distal intestine of 15 dpf Tra-/-/Nac-/- larvae fed HCD for 10 days.
