# Supplementary material for: Dietary cholesterol directly induces acute inflammasome-dependent intestinal inflammation
Source: Nat Commun. 2014 Dec 23;5:5864. doi: 10.1038/ncomms6864 (PMC4284652; doi:10.1038/ncomms6864)
Supplement: Supplementary Figures — 1-13. [file ncomms6864-s1.pdf]

Supplementary Figures 1-13:

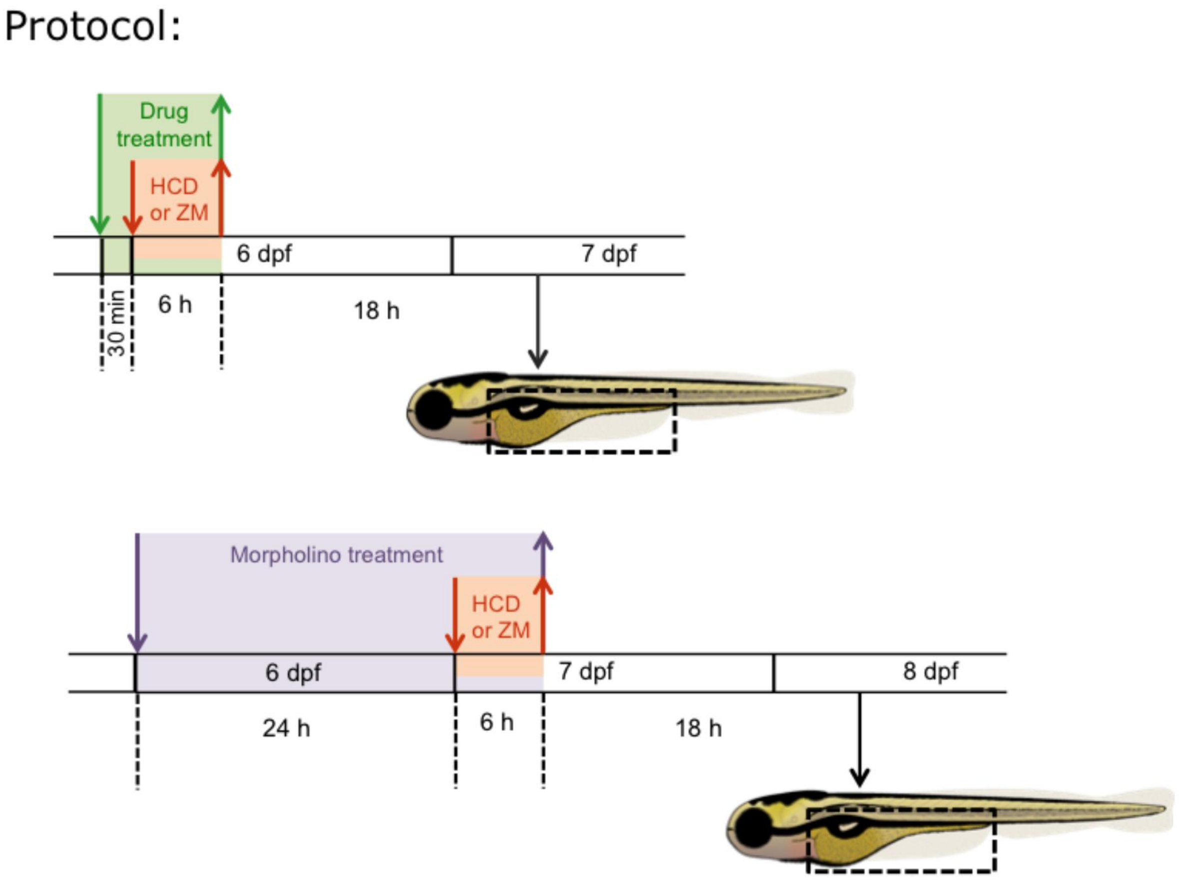

Fig. 1. Schematic representation of the protocol used for experiments. Zebrafish larvae at 6 days post-fertilisation (dpf) were pre-treated with indicated drugs for 30 minutes (min) followed by feeding with ZM control diet or high cholesterol diet (HCD) for 6 hours (h) in the presence of drugs (top panel). Zebrafish larvae (6 dpf) were pre-treated with indicated morpholino oligonucleotides for 24 hours and followed by feeding with ZM control diet or HCD for 6 hours in the presence of morpholinos (bottom panel). The quantification of total cell numbers of indicated immune cells in the intestine were performed at 18 hours post feeding.

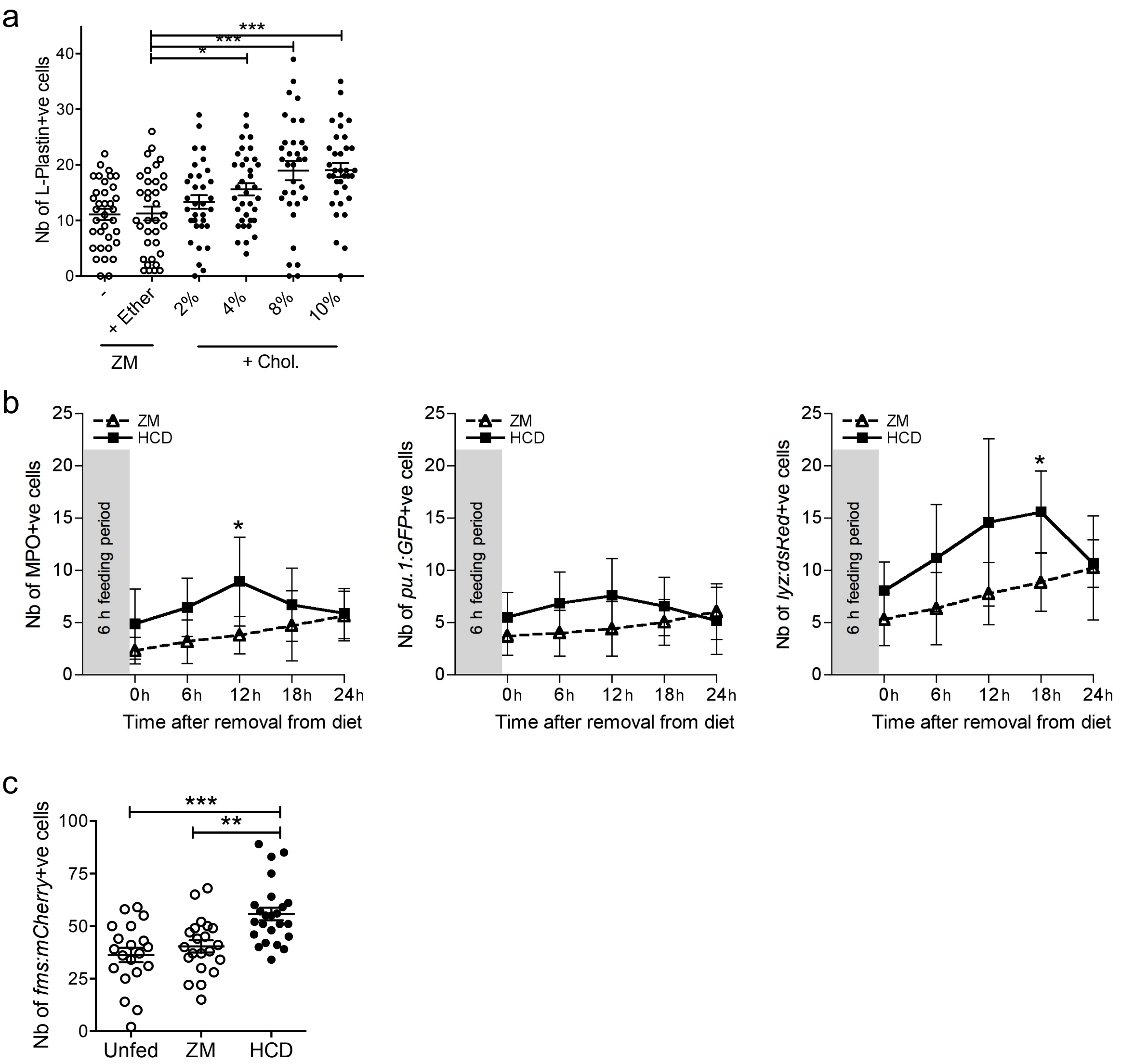

Fig. 2. HCD induces accumulation of various myeloid cells in the larval intestine.
(a) Total number of L-Plastin+ cells in the intestine of zebrafish larvae at 18 hours following HCD at different indicated concentration for 6 hours. n≥35. Errors bars are s.e.m. One-way Anova. **(b)** Total number of MPO+, GFP+ and dsRed+ cells in 6 dpf larvae WT, *Tg(pu.1:GFP)* or *Tg(lyz:dsRed)* 6 dpf larvae fed control (ZM, black triangle) or HCD (black squares) for 6 h in the intestine after 18 h. n≥15. Error bars represent 95% confidence intervals. Two-way Anova. **(c)** Total number of mCherry+ macrophages in *Tg(fms:mCherry)* larvae at 18 hours following feeding with HCD, ZM or unfed. One-way Anova. Errors bars are s.e.m. * p < 0.05, ** p < 0.01, *** < 0.001.

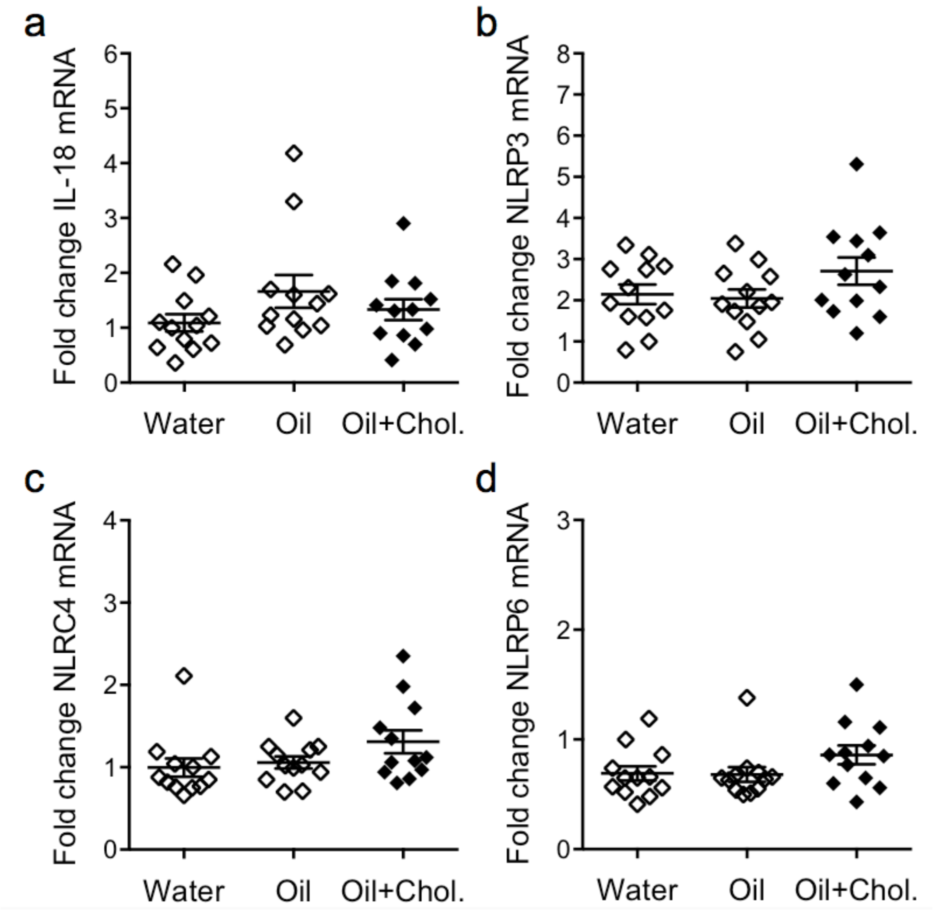

Fig. 3. No change in expression of IL-18, NLRP3, NLRC4 or NLRP6 mRNA following HCD.

(a-d) Fold change of **(a)** IL-18, **(b)** NLRP3, **(c)** NLRC4 and **(d)** NLRP6 mRNA assessed by qRT-PCR in the small intestine (ileum) of Balb/C mice 12 h after gavage with butter, oil or oil+cholesterol. Relative expression values were normalised to 18S and presented relative to one control sample. Each dot represents one individual mouse pooled from four experiments (n=12). Errors bars are s.e.m. (a, c, d) Kruskal Wallis test. (b) One-way Anova.

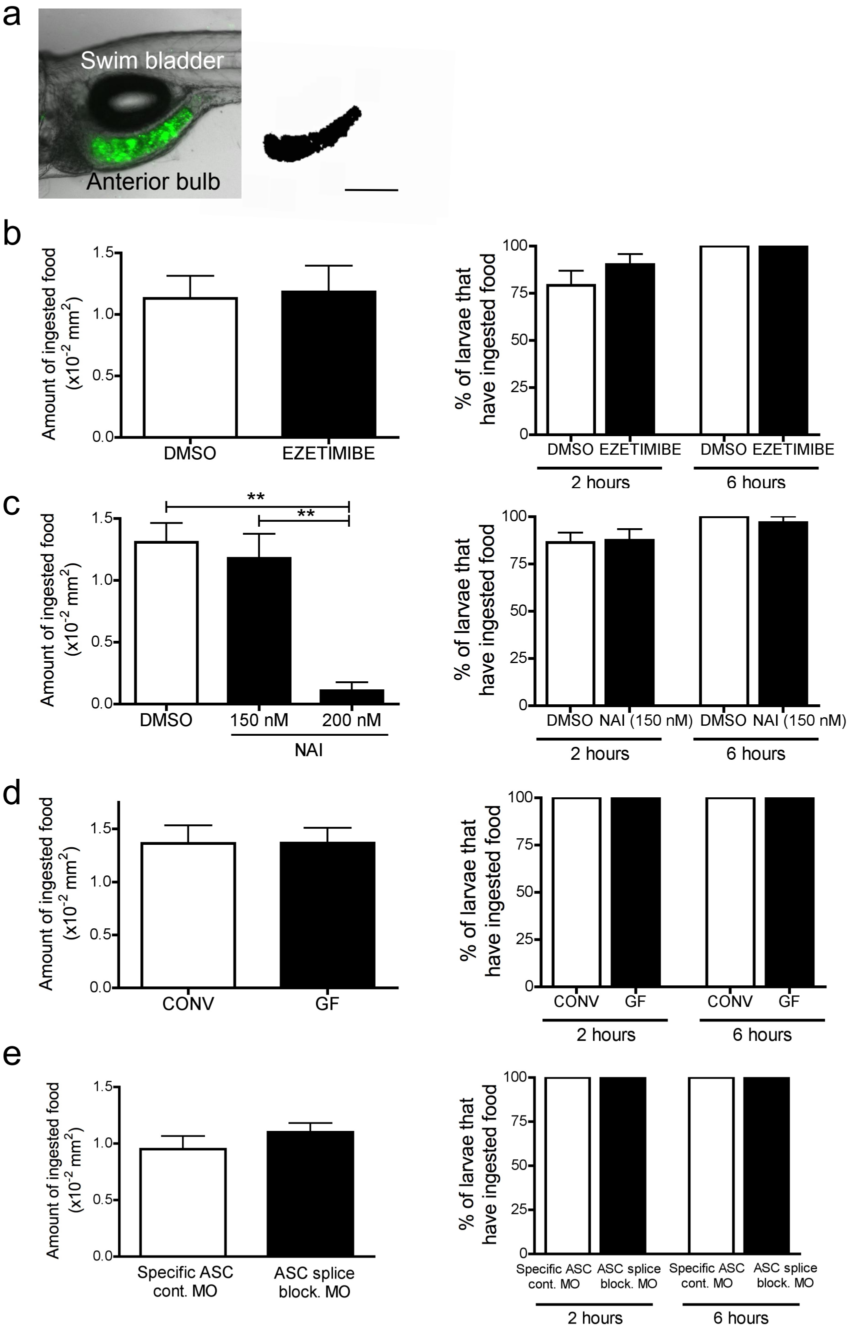

Fig. 4. Food uptake and ability to ingest food is not impaired following ezetimibe, NAI or MO treatment or under germ free conditions

(a) Representative fluorescent image of zebrafish larvae fed HCD supplemented with fluorescent microspheres for 2 hours. The amount of ingested food (HCD) was assessed by quantifying the thresholded binary patterns of the area of the anterior bulb filled with fluorescent microspheres-labelled HCD (shown on the right). Scale bar: 100 µm **(b-e)** Amount of ingested food (HCD) at 2 hours (left panels) and % of larvae that have ingested food by 2 hours and at the end of the 6 hour feeding period (right panels) following treatment with **(b)** 25 µM of ezetimibe (n≥29) or **(c)** 150 or 200 nM (indicated) of NAI (n≥32) compared to DMSO control; in **(d)** germ-free conditions compared to conventional conditions and fed sterile HCD (n≥30); and in **(e)** following treatment with ASC splice blocking MO (20 µM) compared to a specific ASC control MO (n≥19). Data is pooled from two experiments. Errors bars are s.e.m. (b, c right panel, d and e) Mann-Whitney test. (c left panel) One-way Anova. ** $p < 0.01$

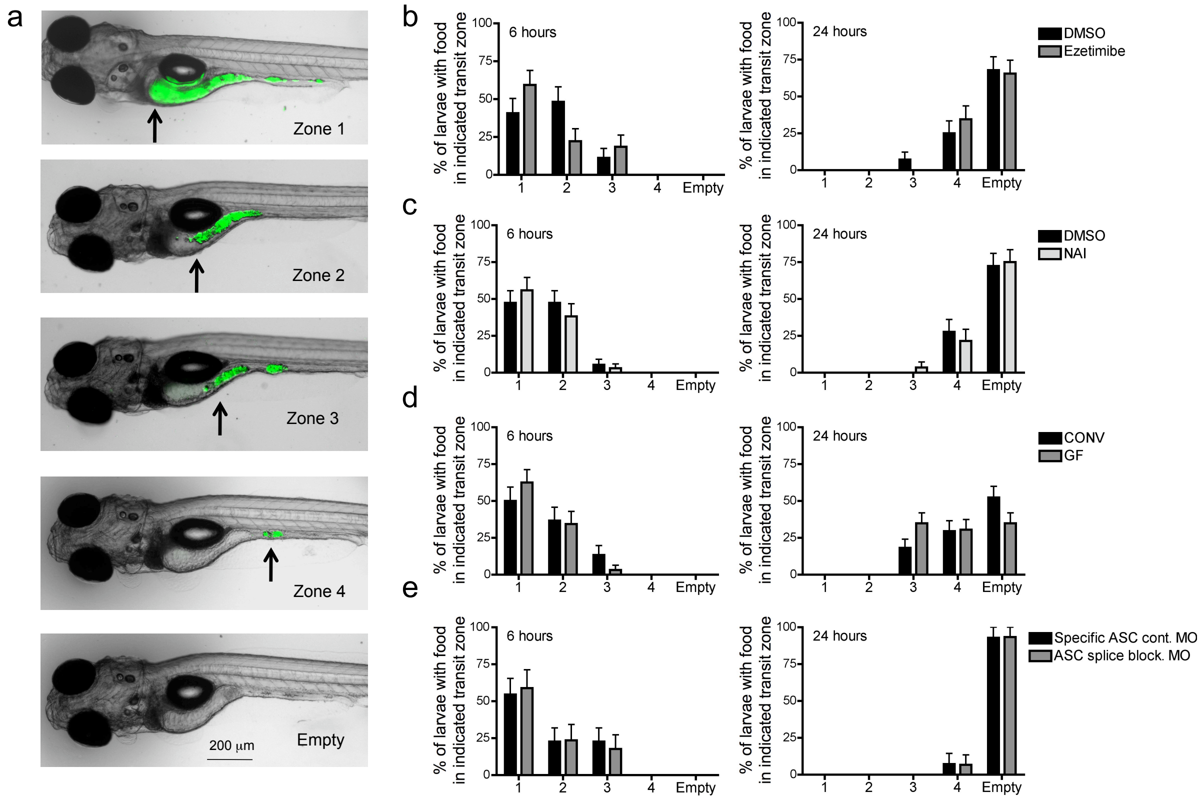

Fig. 5. Food transit is not impaired following ezetimibe, NAI or MO treatment or under germ free conditions

(a) Example of food transit through the intestine using HCD supplemented with fluorescent microspheres over time. Transit zones according to Field *et al.*[52] are indicated. Food transit was assessed by recording the most anterior position of fluorescent microspheres-tagged HCD using live stereomicroscopy. Scale bar: 200 µm. **(b-e)** Quantification of larvae with ingested HCD in intestinal transit zones at 6 hours (left panel) and 24 hours (right panel) following treatment with **(b)** 25 µM of ezetimibe (n≥27) or **(c)** 150 nM of NAI (n≥32) compared to DMSO control; in **(d)** germ-free conditions compared to conventional conditions and fed sterile HCD (n≥29); and in **(e)** following treatment with 20 µM of ASC splice blocking MO compared to a specific ASC control MO (n≥19). Data is pooled from two experiments. Errors bars are s.e.m. Mann-Whitney test for each transit zone. There was no statistical difference between either treatment groups.

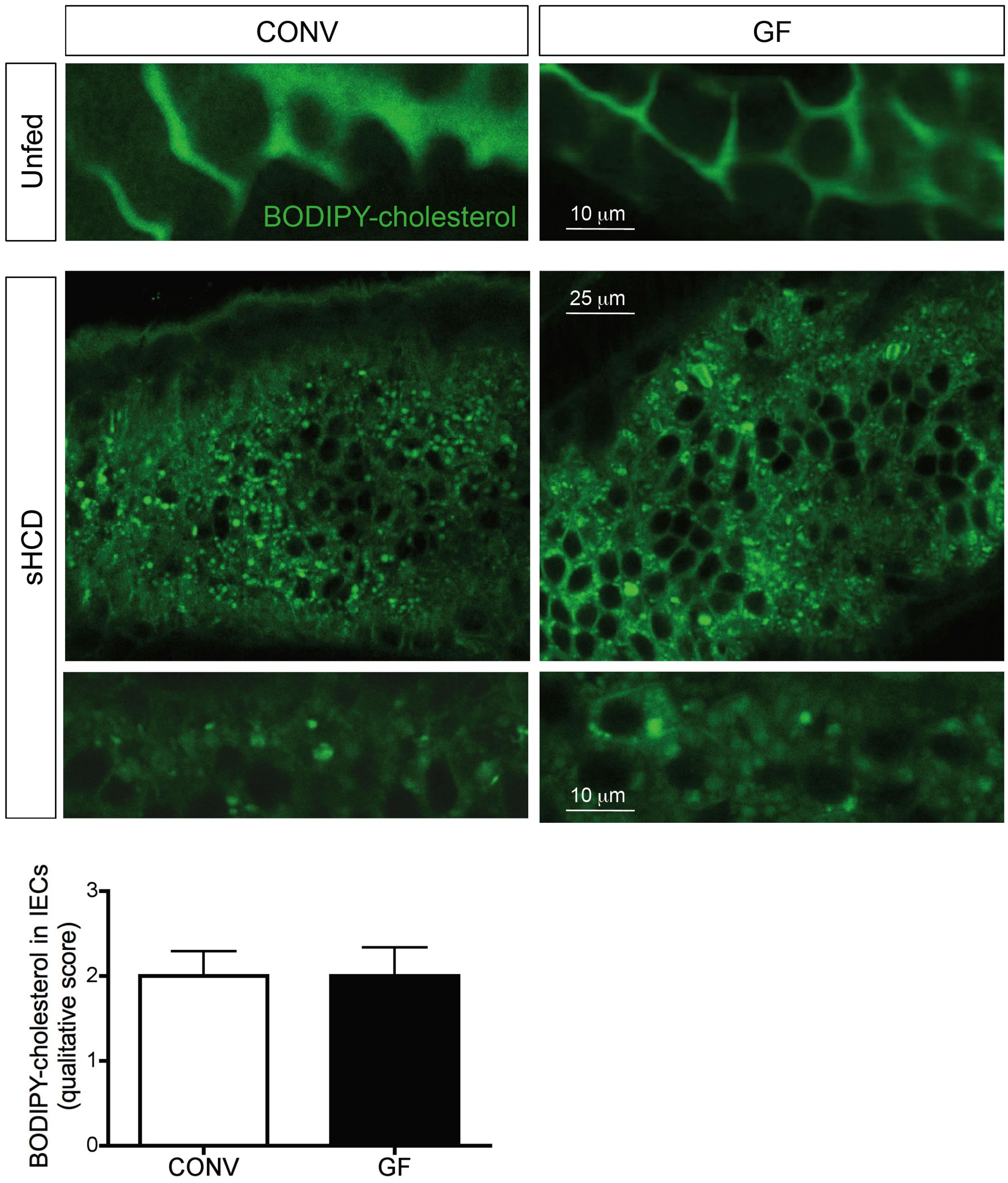

Fig. 6. Cholesterol uptake by IECs is not impaired in germ free conditions
Confocal images (63x) of live intestinal epithelial cells of larvae raised under conventional (left panels) or germ-free (right panels) conditions and fed sterile HCD (middle and bottom row) or left unfed (top row) and treated with BODIPY-cholesterol. BODIPY-cholesterol uptake into intestinal epithelial cells (IECs) requires food, but is not impaired in germ-free conditions. Images were analysed blind for cytoplasmic fluorescence. The presence of strong fluorescence intensity and an abundant signal was scored with 3 and the absence of a signal was scored with 0. Bar chart shows mean + s.e.m., pooled from two independent experiments (n=9). Scale bars: 20 µm. Mann Whitney test. n.s. non-significant.

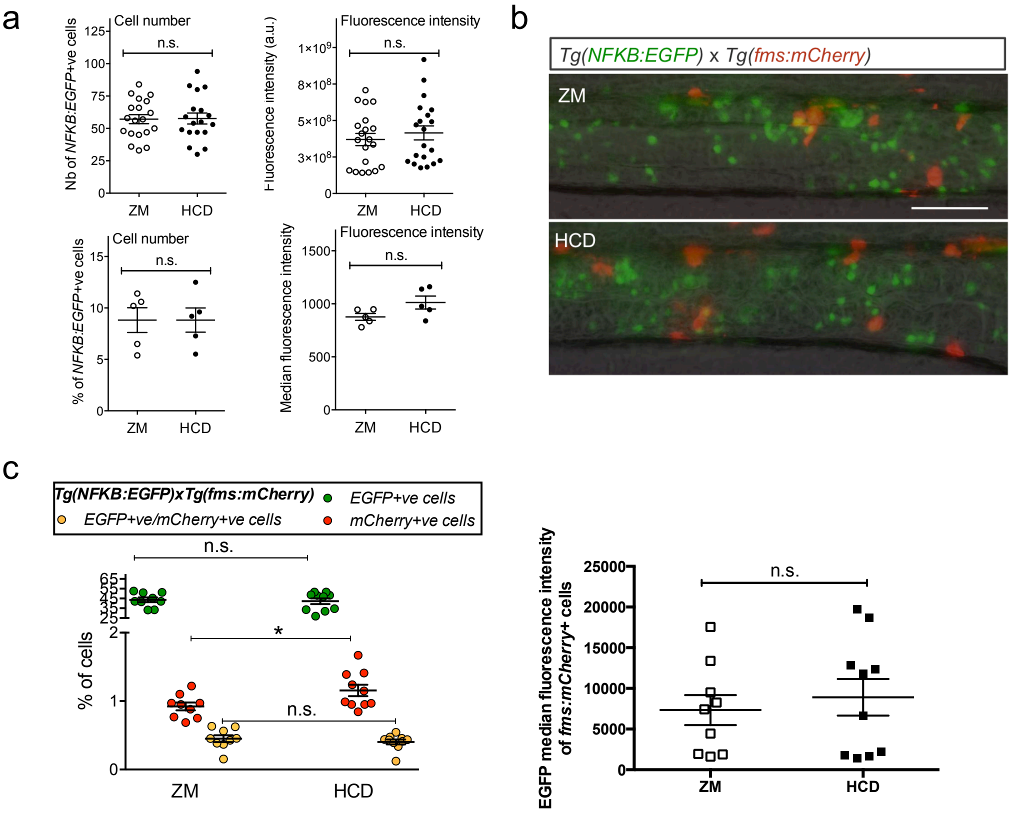

Fig. 7. HCD does not further activate NF-κB expression in the intestine of *Tg(NFkB:EGFP)*.

In all figures, analysis was performed at the peak of inflammation at 6 hours feeding + 18 hours. **(a)** EGFP+ cells and fluorescence intensity (top row) and percentage and median fluorescence intensity of EGFP+ cells (bottom row) in the intestine of *Tg(NFkB:EGFP)* larvae analysed by ImageJ (top row) and flow cytometry (bottom row), respectively. Individual larval intestines were analysed when using image analysis, n≥18, pooled from two experiments (top row) and 3 larval intestines were pooled for analysis by flow cytometry, n≥5, pooled from two experiments. Two-tailed t-test and Mann Whitney test. **(b)** Representative images of intestine of *Tg(NFkB:EGFP xTg(fms:mCherry)* larvae at 18 hours following ZM or HCD for 6 hours. Scale bar: 50 µm. Two-tailed t-test. **(c)** Percentage (left panel) of EGFP single positive, mCherry single positive and EGFP/mCherry double positive cells in the intestine of *Tg(NFkB:EGFP)xTg(fms:mCherry)* analysed by flow cytometry. Right panel shows EGFP median fluorescence intensity of mCherry positive cells. 5 larval intestines were pooled for analysis by flow cytometry, n≥8, pooled from two experiments. Errors bars are s.e.m. Mann Whitney test. * $p < 0.05$. n.s. non-significant

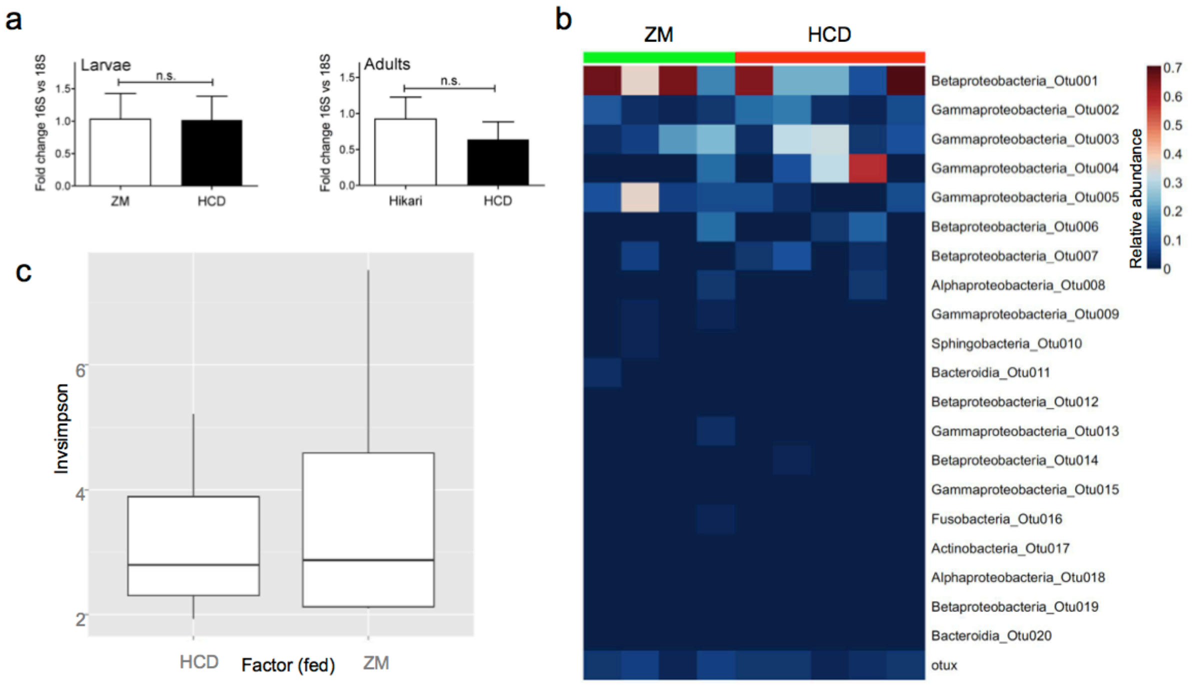

Fig. 8. Analysis of the gut microbiome load and composition shows no effect of HCD. **(a)** Fold change in bacterial load: ribosomal RNA 16S versus 18S in intestines from larvae (pool of 20) or adult (individual) zebrafish. Each bar represents n≥6 pooled from three experiments. Mann Whitney test. n.s. non-significant. **(b)** Heatmap of main bacterial OTUs in larval intestines (each column represents the bacterial profile of 20 pooled larval intestines), n = 4 for ZM, n = 5 for HCD. No statistical differences were noted at any taxonomic level between the two groups. **(c)** Box plot of the inverse Simpson's alpha index of diversity for the ZM and HCD fed groups, there was no statistical difference between the groups. In all figures, analysis was performed at the peak of inflammation at 18 hours following ZM or HCD for 6 hours.

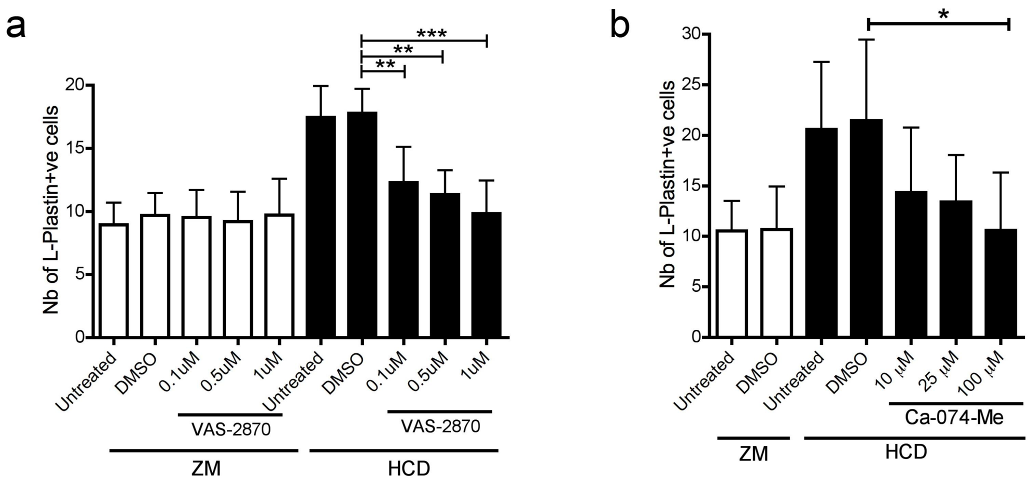

Fig. 9. Titration of NADPH oxidase and Cathepsin B inhibitors.
(a) Total number of L-Plastin+ cells in the intestine of 6 dpf embryos pretreated with various concentrations of VAS-2870 for 30 min or DMSO control and fed HCD or ZM control diet after 18 h. n≥33, pooled from two experiments. Kruskal Wallis test. **(b)** Total number of L-plastin+ cells in the intestine of 6 dpf embryos pre-treated with various concentrations of Ca-074-Me for 30 min or DMSO control and fed HCD or ZM control diet after 18 h. n≥15, one experiment. Error bars represent 95% confidence intervals. Kruskal Wallis test. *** $p < 0.001$, ** $p < 0.01$ and * $p < 0.05$.

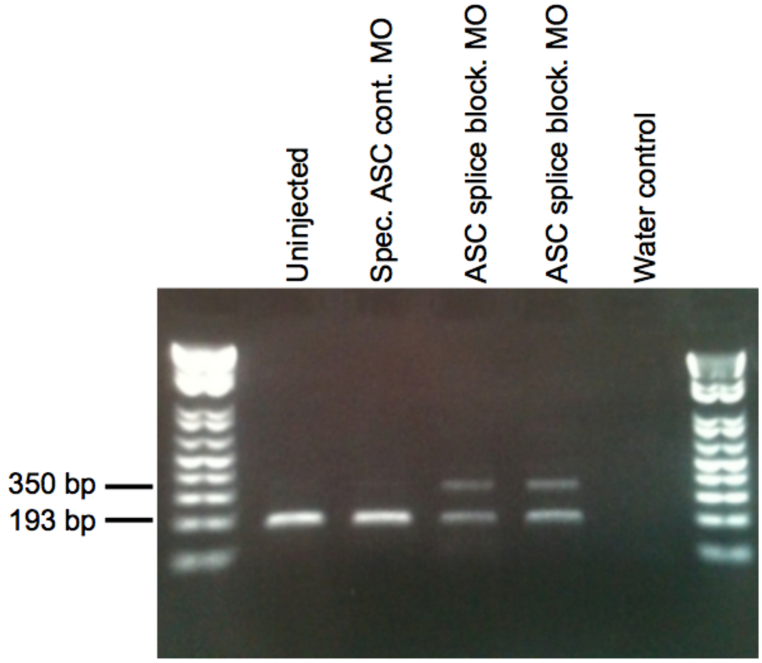

Fig. 10. Unspliced ASC mRNA expression following injection with ASC Splice Blocking Morpholino (MO). PCR products from spliced (193 bp) or unspliced (350 bp) ASC (PYCARD) mRNA of embryos injected with a specific ASC control (for splice blocking) MO, ASC splice blocking MO or left uninjected. RNA was extracted at day 5 from pools of 4 embryos in each group. 1 Kb Plus Ladder loaded in the first and the last lane.

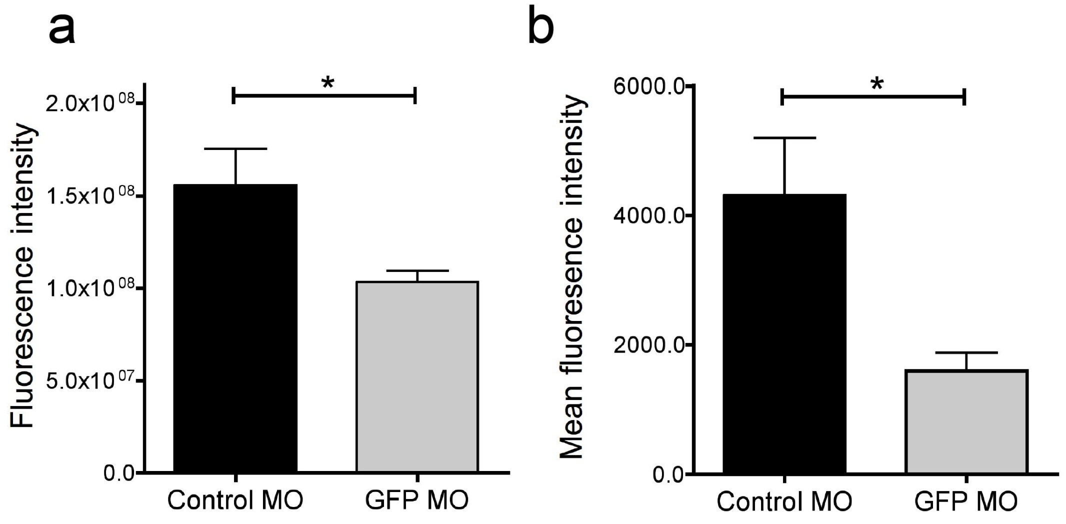

Fig. 11. Treatment with GFP morpholino induces GFP knock-down in the intestine of *Tg(ubi:EGFP)* zebrafish larvae

(a) Fluorescence intensity quantified with ImageJ in the intestine of 8 dpf *Tg(ubi:EGFP)* zebrafish larvae treated for 48 hours with 20 µM GFP MO or control MO. n≥32, pooled from four experimental replicates. **(b)** Mean fluorescence intensity of cell suspension isolated from the intestine of 8 dpf *Tg(ubi:EGFP)* zebrafish larvae treated for 48 hours with 20 µM GFP MO or control MO and analysed by flow cytometry. n≥31, pooled from four experiments. Error bars represent s.e.m. Mann-Whitney test. * $p < 0.05$.

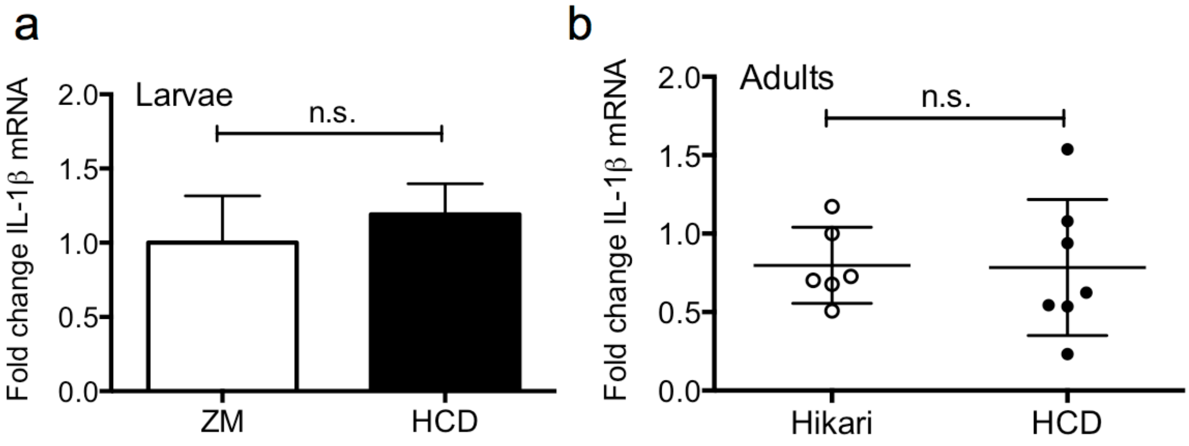

Fig. 12. IL-1β transcript levels in larval and adult zebrafish intestines following HCD. **(a&b)** Fold change of IL-1β mRNA assessed by qRT-PCR in **(a)** larval intestines (pool of 20 intestines per sample, n = 3) and **(b)** adult intestines (individual, n≥6) following HCD or control diet for 6 hours. Relative expression values were normalised to 18S and presented relative to one control sample. Error bars are s.d. Mann Whitney test. n.s.= non significant.

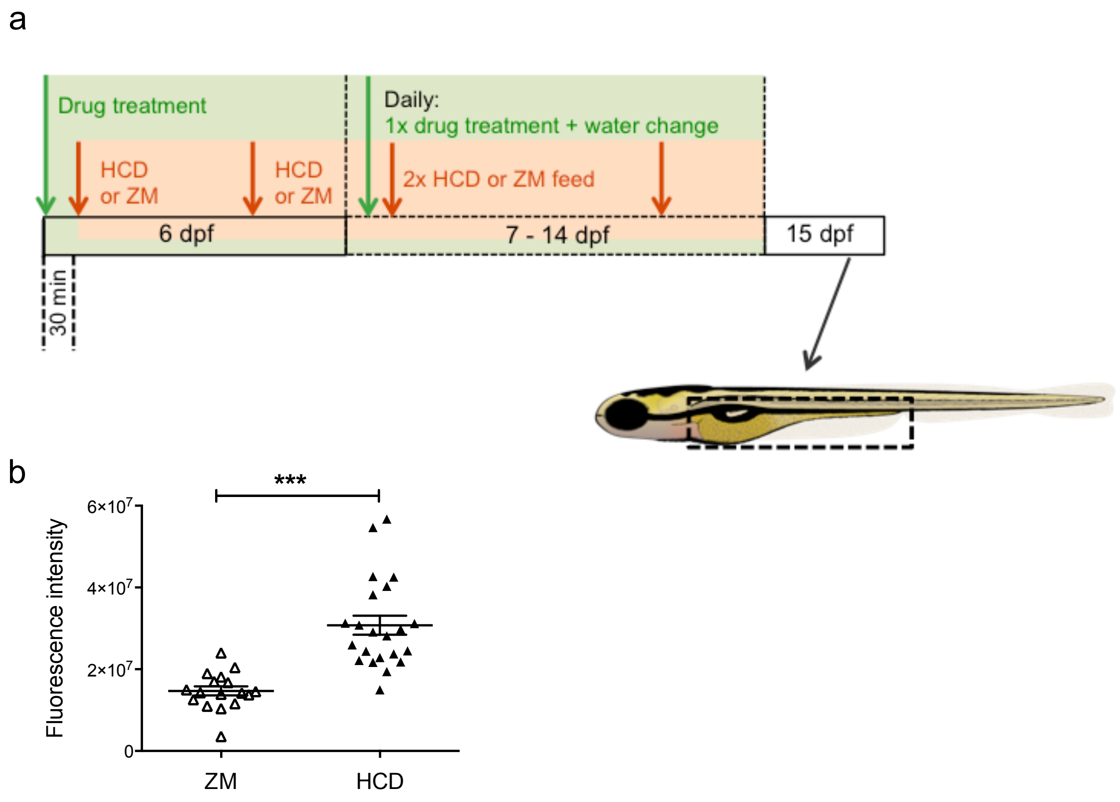

Fig. 13. Fatty deposits in caudal vein are observed following extended HCD feeding. **(a)** Schematic representation of the protocol used for 10 day feeding and drug treatment experiments. Zebrafish larvae at 6 days post-fertilisation (dpf) were pre-treated with indicated drugs for 30 minutes (min) followed by feeding twice daily with ZM control diet or high cholesterol diet (HCD) for 10 days in the presence of drugs and fish were analysed at 15 dpf. **(b)** Fatty deposits in caudal vein by fluorescence intensity measurements using ImageJ in *Tra*$^{-/-}$/*Nac*$^{-/-}$ fish fed HCD or ZM control for 10 days supplemented with fluorescent BODIPY® cholesteryl ester. n≥17, one representative experiment of three. Error bars represent s.e.m. Two-tailed t-test. *** $p < 0.001$.
